# Yeast casein kinase 2 governs morphology, biofilm formation, cell wall integrity, and host cell damage of *Candida albicans*

**DOI:** 10.1371/journal.pone.0187721

**Published:** 2017-11-06

**Authors:** Sook-In Jung, Natalie Rodriguez, Jihyun Irrizary, Karl Liboro, Thania Bogarin, Marlene Macias, Edward Eivers, Edith Porter, Scott G. Filler, Hyunsook Park

**Affiliations:** 1 Division of Infectious Diseases, Chonnam National University Medical School, Gwangju, South Korea; 2 Department of Biological Sciences, California State University, Los Angeles, Los Angeles, California, United States of America; 3 Division of Infectious Diseases, Department of Medicine, Los Angeles Biomedical Research Institute at Harbor-UCLA Medical Center, Torrance, California, United States of America; Louisiana State University, UNITED STATES

## Abstract

The regulatory networks governing morphogenesis of a pleomorphic fungus, *Candida albicans* are extremely complex and remain to be completely elucidated. This study investigated the function of *C*. *albicans* yeast casein kinase 2 (CaYck2p). The *yck2Δ/yck2Δ* strain displayed constitutive pseudohyphae in both yeast and hyphal growth conditions, and formed enhanced biofilm under non-biofilm inducing condition. This finding was further supported by gene expression analysis of the *yck2Δ/yck2Δ* strain which showed significant upregulation of *UME6*, a key transcriptional regulator of hyphal transition and biofilm formation, and cell wall protein genes *ALS3*, *HWP1*, and *SUN41*, all of which are associated with morphogenesis and biofilm architecture. The *yck2Δ/yck2Δ* strain was hypersensitive to cell wall damaging agents and had increased compensatory chitin deposition in the cell wall accompanied by an upregulation of the expression of the chitin synthase genes, *CHS2*, *CHS3*, and *CHS8*. Absence of CaYck2p also affected fungal-host interaction; the *yck2Δ/yck2Δ* strain had significantly reduced ability to damage host cells. However, the *yck2*Δ/*yck2*Δ strain had wild-type susceptibility to cyclosporine and FK506, suggesting that CaYck2p functions independently from the Ca+/calcineurin pathway. Thus, in *C*. *albicans*, Yck2p is a multifunctional kinase that governs morphogenesis, biofilm formation, cell wall integrity, and host cell interactions.

## Introduction

*Candida albicans* is a part of the normal microbiota of human hosts. As an opportunistic pathogen, it can cause life-threatening infections under immunocompromised conditions [1, 2]. Its ability to switch morphologies in various host environments is a key virulence trait [3–8]. Morphologic transition is regulated by complex pathways [9–12], and regulatory cross talk among the signaling pathways plays a significant role in governing morphogenesis in response to various environmental cues [13, 14]. Morphogenesis is closely associated with biofilm formation. The *C*. *albicans* biofilm architecture contains a mixture of yeast and hyphal cells in structured layers [15–18], and is largely related to its dimorphic nature in response to environmental factors such as temperature, pH, and media composition [19–21]. Previous studies suggest that many genes involved in hyphal formation are also tied to genes governing biofilm formation [16, 22–24]. It is also known that cell wall biogenesis directly contributes to the morphogenesis and pathogenicity of *C*. *albicans*, as the cell wall structure dynamically changes during progression of the cell cycle [25]. Thus, it is necessary to understand the interconnections amongst the various regulatory pathways that govern cell wall biogenesis and morphogenesis for therapeutic and prophylactic interventions.

CaYck2p is one of the fungal homologs of the casein kinase 1 (CK1) family of highly conserved serine/threonine kinases that are present in most eukaryotic organisms [26]. In higher eukaryotes, the CK1 family is part of the Wnt signaling pathway [27] and regulates cellular differentiation [28], membrane trafficking [29], DNA damage response [30], and circadian rhythms [28]. The *Candida* genome project has revealed three CK1 paralogs (*YCK2*, *YCK3*, and *HRR25*) that share amino acid similarity to other fungal CK1s, such as ScYck1p and ScYck2p in *Saccharomyces cerevisiae* (S1 Fig). In *S*. *cerevisiae*, CK1 members are functionally related, governing the cell cycle, morphology, and cell wall integrity [26, 31, 32]. Furthermore, in *S*. *cerevisiae*, ScYck1p and ScYck2p are known to regulate endocytosis, cell morphogenesis, mRNA localization, and nutrient sensing [26, 31, 33, 34]. Based on its sequence similarity to ScYck1p and ScYck2p, *C*. *albicans* Yck2p (CaYck2p) is predicted to play a role in nutrient sensing [34, 35]. A recent large-scale screening of a *C*. *albicans* protein kinase insertion library revealed that CaYck2p and CaYck3p are critical for cell wall regulation [36]. Yet, further analysis of individual CK1s in *C*. *albicans* has not been performed.

Our previous study discovered that transcription of the *YCK2* gene was significantly upregulated when *C*. *albicans* interacted with host endothelial cells, and analysis of a *yck2* insertion mutant suggested that CaYck2p may govern host cell interactions [37]. However, a limitation of this work was that it analyzed a *yck2* mutant in which the insertion cassette was integrated at bp 1130 in the *YCK2* open reading frame. As a result, the truncated 376 amino acid-protein likely retained partial function (S1 Fig). To better elucidate the function of CaYck2p in *C*. *albicans* and to determine the functional conservation among members of the fungal CK1 family, we generated and investigated a homozygous *yck2*Δ/*yck2*Δ strain in which the entire protein coding region of the *YCK2* was deleted. We identified a new function of CaYck2p in governing cell growth and hyphal transition, *in vitro* biofilm formation, and cell wall biogenesis. We also uncovered several potential downstream molecules affected by CaYck2p that govern these cellular processes of *C*. *albicans*.

## Materials and methods

### Strains and media

All *C*. *albicans* strains constructed and used in this study are listed in S1 Table. Strains were maintained on YPD agar (1% yeast extract (Difco), 2% peptone (Difco) and 2% glucose) at 30°C. Synthetic complete medium [SC, 0.67% yeast nitrogen base without amino acids (Difco), 0.065% synthetic complete supplement mixture without histidine, arginine, and uridine (Qbiogene), 2% glucose, and 2% agar, supplemented with 100 μg/ml arginine, 50 μg/ml histidine, and 20 μg/ml uridine as needed] was used for strain construction and screening. YPD, RPMI 1640 (Hyclone), Spider (1% nutrient broth, 1% mannitol, 0.2% K_2_PO_4_) and YPGly (YP, 2% glycerol) broth were used for testing hyphal induction and biofilm formation.

### Strains construction

The *yck2*Δ/*yck2*Δ strain was derived from *C*. *albicans* BWP17 by successive transformation with *ARG4* and *HIS1* deletion cassettes generated by PCR using the primers *YCK2* KO-5 and *YCK2* KO-3 (S2 Table) [38]. Transformants with *ARG4* and *HIS1* markers were selected on SC medium supplemented with 50 μg/ml histidine and 20 μg/ml uridine amino acids, respectively. The resulting *yck2*Δ/*yck2*Δ strain was subsequently transformed with *Not* I (Promega) and *Pst* I (Promega) digested 3.8 Kb pBSK-URA3 to re-integrate *URA3* at its native locus as previously described [39]. To construct the *YCK2* complemented strain (*yck2*Δ/*yck2*Δ::*YCK2*), a 2.85 Kb fragment containing *YCK2* was amplified from SC5314 genomic DNA with the primers *YCK2* Comp-5 and *YCK2* Comp-3 by high fidelity PCR (Takara). This PCR product was then cloned into *Not* I digested pBSK-URA3 [39]. The resulting construct was linearized with *Nru* I for direct integration at the *URA3* locus of the *yck2*Δ/*yck2*Δ strain. Targeted deletion of *YCK2* was confirmed by whole cell PCR with the primers, *YCK2* Confirm-5 and *YCK2* Confirm-3.

### Morphology analysis

To induce hyphal growth of *C*. *albicans*, the cells were grown in 3 ml YPD medium for overnight. The blastopores were then harvested by centrifugation for 10 min at 1000 × *g* and washed twice with 1X phosphate buffered saline (PBS). The final concentration of 3 × 10^6^ cells/ml blastospores were added into RPMI 1640 medium (Hyclone) and incubated at 37°C for 3 h. Then 10 μl aliquots of the final resuspension was added to glass slides and mixed with 10 μl Vectashield Antifade Mounting Media (Vector Laboratories) for microscopic evaluation under a Zeiss Apotome microscope (Zeiss).

### Biofilm assay

The extent of biofilm formation by the various *C*. *albicans* strains was measured by a 96-well microtiter plate biofilm assay [40]. Briefly, the overnight grown cells were washed with PBS and then diluted to 10^7^ cells/ml, in various media, and 100 μl of each suspension was transferred in quadruplicate into 96-well polystyrene, round-bottom microtiter plates (Thermo Fisher Scientific). Medium without *C*. *albicans* cells was used as a baseline control. The extent of biofilm was quantified after 24 h by crystal violet staining. First, the wells were washed twice with 200 μl of PBS and then dried for 45 minutes at room temperature. Then, 110 μl of 1% crystal violet solution (Thermo Fisher Scientific) was added and incubated for 45 minutes at room temperature. The wells were then washed four times with 350 μl sterile water to remove the unbound crystal violet. Finally, the biofilm-associated crystal violet was solubilized with 110 μl of 95% ethanol (Sigma-Aldrich) at room temperature for 45 min. Subsequently, 100 μl was transferred into a new 96 well plate for reading at 570 nm (VictorX, Perkin Elmer, Waltham, MA).

### Susceptibility testing

A spot dilution assay was used to test the susceptibility of various *C*. *albicans* strains to the cell membrane and cell wall stressors, as previously described [41]. Briefly, the cells were grown for overnight in YPD at 30°C and counted using a hemocytometer. Serial 10-fold dilutions of the strains in 5 μl PBS (range 10^5^ to 10^1^ colony forming units (CFU) per spot) were then plated onto YPD agar containing 300 μg/ml congo red, 10 μg/ml calcofluor white, 2 mg/ml protamine sulfate, 0.1% sodium dodecyl sulfate, 10 μg/ml cyclosporine A, 5 μg/ml FK506 and 5 μg/ml pyrvinium pamoate, respectively, and incubated at 30°C for 48 h. The plates were photographed in UVP Gel Doc-It system (UVP, CA).

The minimum Inhibitory Concentrations (MIC) of various *C*. *albican* strains to fluconazole and caspofungin were tested using the broth microdilution method of the CLSI according to document M27-A3 [42]. MIC values were determined after 24 h and 48 h of incubation.

### Calcofluor white staining and fluorescent microscopy

The *C*. *albicans* strains were grown to log phase in YPD medium at 30°C and then further incubated with 0.05% calcofluor white solution (Sigma) for 5 min, after which they were washed once and resuspended in PBS. To mount the cells, 10 μl of final resuspension was added to the glass slide and mixed with 10 μl of Vectashield Antifade Mounting Media (Vector Laboratories). To compare the degree of the calcofluor white fluorescent signal, the UV laser intensity and the exposure time were fixed at the same level for all observation. The images were photographed with 63X magnification under a Zeiss Apotome microscope.

### Host cell damage assay

Human umbilical vein endothelial cells (HUVECs) were harvested as previously described [43]. The cells were maintained in M-199 medium (Gibco) supplemented with 10% fetal bovine serum, 10% bovine calf serum, 2 mM L-glutamine, 100 IU/ml penicillin, and 100 μg/ml streptomycin. The immortalized oral mucosal keratinocytes (OKF6/TERT) were kindly provided from Dr. Rheinwald [44] and the immortalized vaginal epithelial cells (VK2/E6E7) were purchased from American Type Culture Collection (ATCC) [45]. The immortalized cells were maintained in keratinocyte serum-free medium (Gibco) supplemented with 50 μg/ml bovine pituitary extract, 0.1 ng/ml epidermal growth factor, 100 U/ml penicillin, and 100 μg/ml streptomycin [44, 45]. All cells were grown at 37°C in a humidified environment containing 5% CO_2_. The extent of damage caused by various *C*. *albicans* strains was measured using a ^51^Cr release assay as described previously [46]. The host cells were grown in a 96-well tissue culture plate with detachable wells and incubated overnight with 6 μCi Na_2_^51^CrO_4_ (MP Biomedicals) per well. The following day, the unincorporated tracer was removed by extensive rinsing. When HUVECs were used, they were infected with 4 × 10^4^ *C*. *albicans* blastospores per well in RPMI 1640. As the epithelial cells are less susceptible to *C*. *albicans*-induced damage, they were infected with 10^5^ *C*. *albicans* blastospores per well in the same medium. To measure the spontaneous release of ^51^Cr, uninfected host cells were exposed to medium alone. The infected host cells were incubated for 3 h and then, the amount of ^51^Cr released into the medium and retained by the cells was determined by liquid scintillation counter 6500 (Beckman Coulter).

### Quantitative real time RT-PCR analysis

*C*. *albicans* cells were grown overnight in 3ml of YPD and adjusted to a 3 × 10^6^ cells/ml subculture. The cells were then incubated for two h in YPD at 30°C for yeast phase growth and in RPMI at 37°C for hyphal growth. Total RNAs were extracted with RiboPure RNA extraction kit following the manufacturer’s protocol (Ambion). cDNAs were synthesized from 1 μg of total RNAs with Retroscript Reverse Transcription Kit (Ambion) following the manufacturer’s protocol. Quantitative real-time PCR was carried out using 5-Prime SYBR green PCR kit (Thermo Fisher Scientific) and Eppendorf Realplex System (Eppendorf) following the manufacturers’ protocol. The primers used in this study are listed in S2 Table. Relative gene expression was calculated by the 2^ΔΔ*CT*^ method [47] using the transcript level of *CaACT1* as the endogenous control [37].

### Statistical analysis

Unless stated otherwise, each experiment was performed in triplicate on at least three separate occasions. Raw data were analyzed with Microsoft Excel software and statistical significance of any differences between experimental groups was determined by ANOVA with Dunnett’s test posthoc analysis. *, **, and *** denotes a *p* value of < 0.05, < 0.001, and < 0.0001, respectively.

## Results

### Yck2p governs morphologic transition of *C*. *albicans*

**Fig 1:**
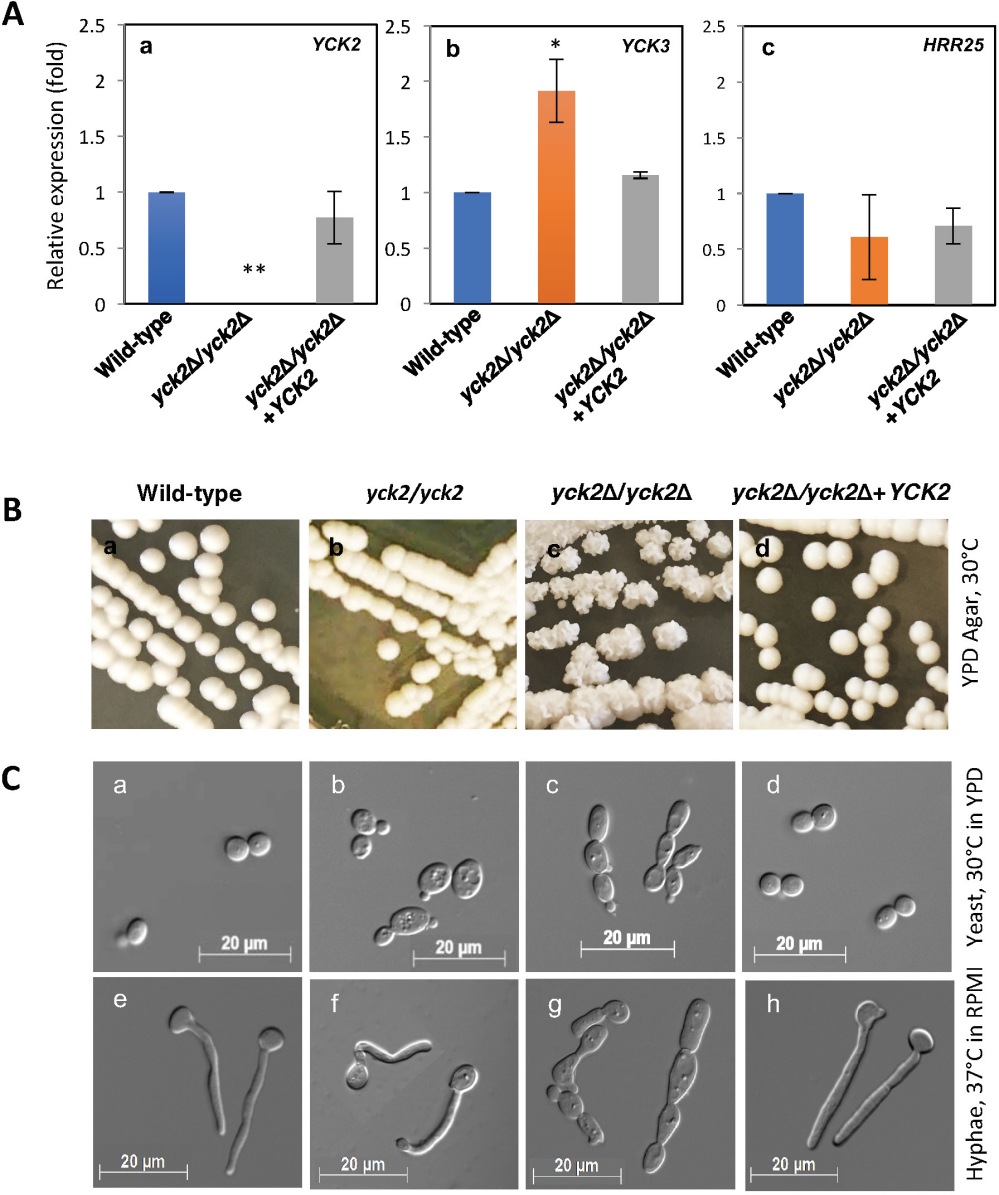
Morphology of the *yck2*Δ/*yck2*Δ strain. **A,** relative mRNA expression of *YCK2* (a), *YCK3* (b), and *HRR25* (c) for the designated strains. Shown are mean ± S.D. of three biological replicates (* p<0.05, ** p<0.001). **B,** colony morphologies of the wild-type (a), the *yck2/yck2* insertion mutant (b), the *yck2*Δ/*yck2*Δ (c), and the *yck2*Δ/*yck2*Δ+*YCK2* complemented (d) strains grown on YPD plate at 30°C for 2 days. **C,** microscopic morphologies of the wild-type (a and e), the *yck2/yck2* insertion mutant (b and f), the *yck2*Δ/*yck2*Δ (c and g), and the complemented (d and h) strains grown in YPD medium for two h at 30°C and in RPMI 1640 medium at 37°C, respectively.

*C*. *albicans* switches morphologies between yeast and hyphae forms in response to various environmental cues. We sought to define the role of CaYck2p during *C*. *albicans* morphogenesis. The *yck2*Δ/*yck2*Δ strain was constructed as summarized in S1 Fig and *YCK2* transcription was found to be below detection (p<0.001, Fig 1A). As there are two additional paralogs of CK1 family, *YCK3* and *HRR25*, their transcription was measured to determine any compensatory upregulation in the absence of *YCK2*. As shown in Fig 1A, *YCK3* transcript levels increased (p<0.05), but *HRR25* transcript levels were not changed in the *yck2*Δ/*yck2*Δ strain compared to the wild-type strain and the *yck2*Δ/*yck2*Δ+*YCK2* complemented strain.

The colony morphology of the *yck2*Δ/*yck2*Δ strain was significantly different from the wild-type strain when grown on YPD agar at 30°C. As shown on Fig 1B, the wild-type strain formed smooth convex colonies. The phenotype of the *yck2* insertion mutant strain (*yck2-Tn7*::*UAU1/yck2-Tn7*::*URA3*, called *yck2* insertion strain hereafter) [37] generated in our previous study was similar to that of the wild-type strain. By contrast, the *yck2*Δ/*yck2*Δ strain formed wrinkled colonies with a rough texture, which were different from both the wild-type and the *yck2* insertion mutant strains. Complementing the *yck2*Δ/*yck2*Δ strain with an intact copy of *YCK2* (*yck2*Δ/*yck2*Δ*+YCK2*, complemented strain hereafter) restored the wild-type colony morphology, indicating that the wrinkled colonies of the *yck2*Δ/*yck2*Δ strain were solely due to the complete loss of functional *YCK2* alleles. In contrast, all strains formed wrinkled colonies on YPD with 10% serum agar at 37°C as shown in S2 Fig. It was noted that the wild-type and the complemented strain formed more flat and fuzzy colonies than the *yck2*Δ/*yck2*Δ strain.

Next, the cells were viewed by microscopy to observe the morphology of the *yck2*Δ/*yck2*Δ strain as shown on Fig 1C. The wild-type strain grew as yeast in YPD medium at 30°C, and grew as hyphae in RPMI 1640 medium at 37°C. Similarly, the *yck2* insertion strain grew as yeast form in YPD medium at 30°C and formed short hyphae in RPMI medium at 37°C, similarly to our previous study [37]. However, the *yck2*Δ/*yck2*Δ strain grew as chains of elongated yeast cells in both YPD medium at 30°C and RPMI medium at 37°C. The chains of elongated cells were reminiscent of pseudohyphae. The complemented strain had wild-type morphology, indicating that the observed pseudohyphal-like morphology of the *yck2*Δ/*yck2*Δ strain was solely due to the loss of functional *YCK2* alleles.

### Yck2p is involved in biofilm formation

While assessing the morphology of the *yck2*Δ*/yck2*Δ strain, we noticed that the cells were aggregated and adhered to the plastic culture tubes in YPD broth at 30°C, which was not usually observed with the wild-type strain. Therefore, we suspected that the *yck2Δ/yck2Δ* strain may have increased biofilm capability. First, we assessed the extent of biofilm by the wild-type strain in various conditions, to determine the effect of media and temperature on biofilm formation (S2 Fig). Growth in RPMI 1640 medium induced the noticeable biofilm formation by the wild-type strain at 30°C, whereas neither YPD nor YPGly media induced biofilm formation at 30°C. The Spider medium induced less biofilm formation by the wild-type strain at 30°C. Thus, we considered the spider medium at 30°C as a moderate biofilm inducing condition. The extent of biofilm formed at 37°C suggest that all media except YPGly at 37°C are biofilm-inducing conditions for the wild-type strain.

**Fig 2:**
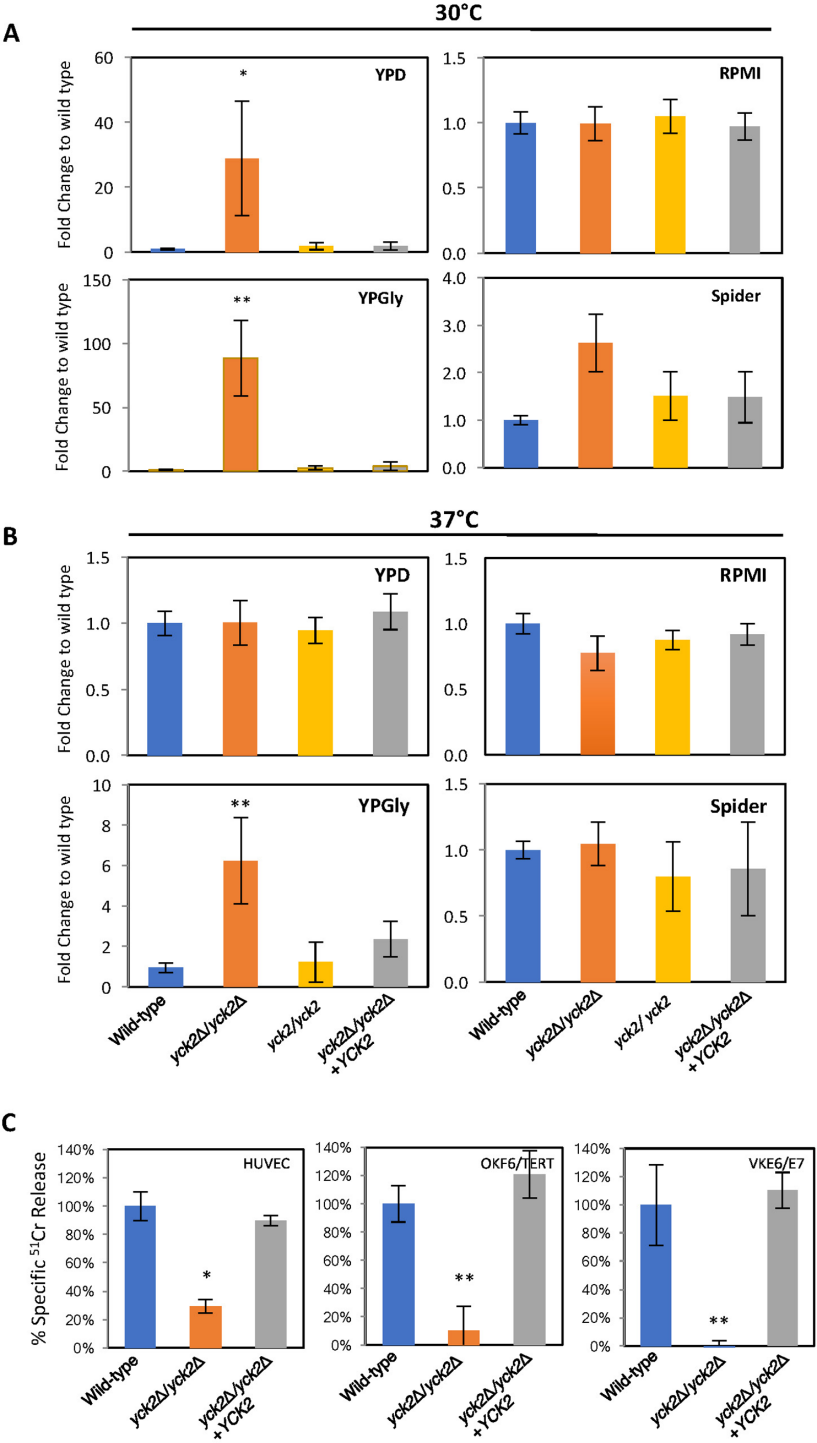
Functional analysis of *YCK2* deletion. **A** and **B,** The extent of biofilm formation by the indicated strains after 48 h in YPD, RPMI1640, YPGly, and Spider media at 30°C (A) and at 37°C (B). (* p<0.001). **C,** The extent of cell damage of endothelial cells (HUVECs), oral epithelial cells (OKF6/TERT), and vaginal epithelial cells (VK2E6/E7) after 180 min co-incubation with the indicated strains. (*, p<0.001 compared to both the wild-type strain and complemented strain).

Next, we measured the extent of biofilm formed by the *yck2*Δ*/yck2*Δ strain in both non-biofilm inducing and biofilm-inducing conditions. Indeed, the *yck2*Δ*/yck2*Δ strain formed significantly increased biofilm in all non-biofilm inducing conditions as compared to the wild-type strain. As shown in Fig 2A, the wild-type strain did not form a biofilm in either YPD or YPGly media at 30°C. In contrast, the *yck2*Δ*/yck2*Δ strain developed 28-fold (p<0.05) and 80-fold (p<0.001) more biofilm in YPD and YPGly, compared to the wild-type strain. The insertion mutant and the complemented strains did not form biofilms in those conditions, similar to the wild-type strain. The extent of biofilm by all strains grown in RPMI 1640 medium at 30°C was comparable, whereas the *yck2*Δ*/yck2*Δ strain formed 2 fold increased biofilm as compared to the wild type strain under spider medium at 30°C. Fig 2B demonstrates that the *yck2*Δ*/yck2*Δ strain formed 6-fold (p<0.001) increased biofilm relative to the wild-type strain in YPGly at 37°C, a non-biofilm inducing condition for the wild-type strain. This result suggests that the *yck2*Δ*/yck2*Δ strain has an enhanced capacity to form biofilms when grown under conditions that induce minimal biofilm formation by the wild-type strain. The *yck2*Δ*/yck2*Δ strain also formed a similar level of biofilm as the wild type did in biofilm inducing condition, suggesting that the *yck2*Δ*/yck2*Δ strain likely formed a constitutive biofilm in all conditions tested for this study.

### Yck2p is required for normal host cell damage by *C*. *albicans*

The ability of *C*. *albicans* to switch morphology greatly affects its ability to damage host cells [3, 10]. We analyzed whether the *yck2*Δ/*yck2*Δ strain with constitutive pseudohyphae was capable of damaging host cells in vitro. As shown in Fig 2C, the *yck2*Δ/*yck2*Δ strain caused significantly less damage to all three types of host cells, human umbilical vein endothelial cells (HUVECs), immortalized oral mucosal keratinocytes (OKF6/TERT), and immortalized vaginal epithelial cells (VK2/E6E7), as compared to the wild-type strain. Complementing the *yck2*Δ/*yck2*Δ strain with a wild-type copy of *YCK2* restored its capacity to damage host cells. This result suggests that CaYck2p is required to damage both endothelial and epithelial cells *in vitro*.

### *YCK2* is involved in cell wall integrity of *C*. *albicans*

**Fig 3:**
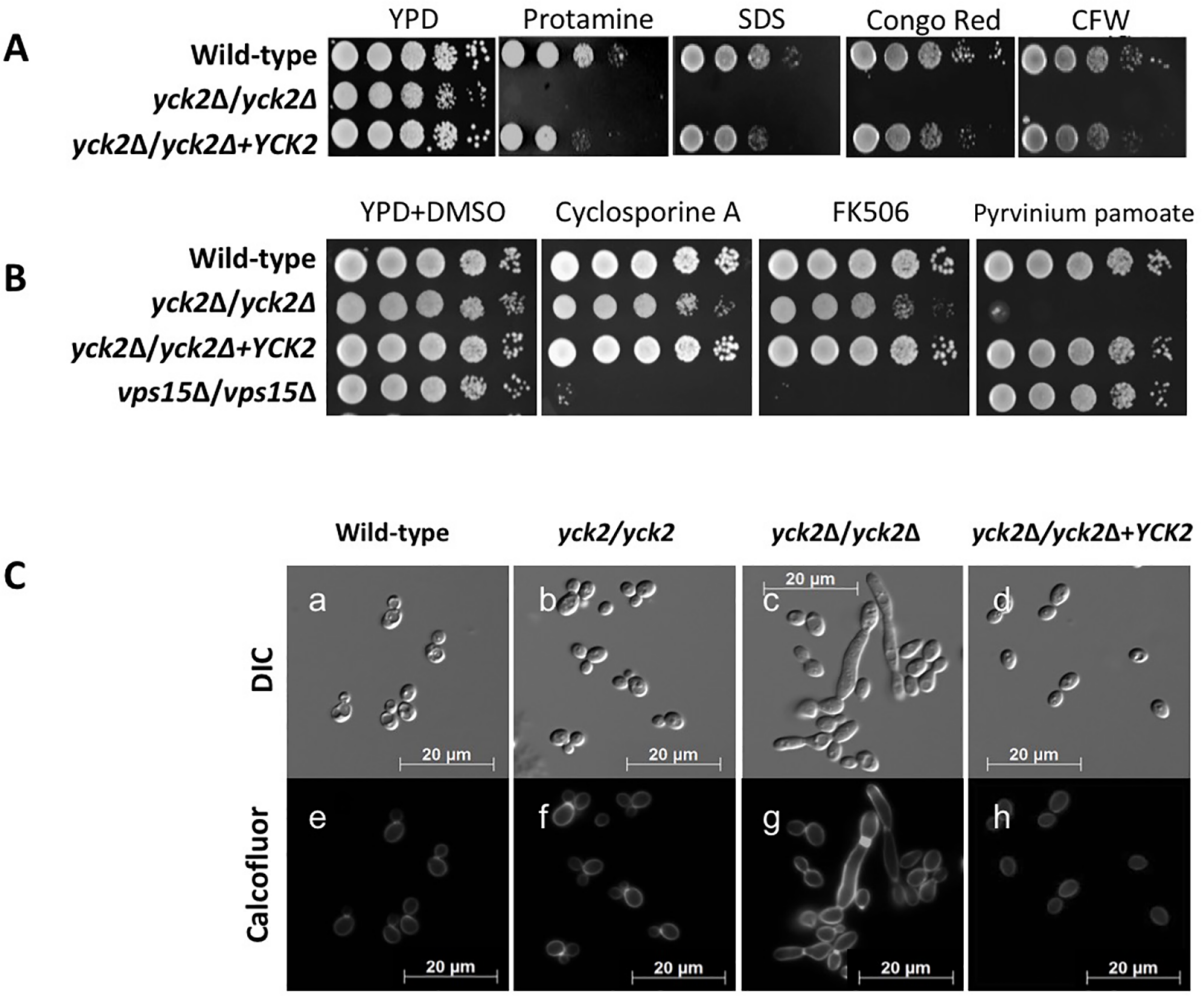
Effect of *YCK2* deletion on cell wall integrity. **A,** susceptibility of the indicated strains to various cell wall stressors, 2 mg/ml protamine sulfate, 0.1% SDS, 300 μg/ml congo red, and 10 μg/ml calcofluor white (CFW) after 48 h at 30°C. **B,** susceptibility of the designated strains to calcineurin inhibitors, 10 μg/ml cyclosporine A and 5 μg/ml FK506 and 5 μg/ml pyrvinium pamoate. Shown are representative images for three independent experiments with the same outcome. **C,** microscopic analysis of chitin deposition in the cell wall of the wild-type (a, e), the insertion mutant (b, f), the *yck2*Δ/*yck2*Δ (c, g), and the complemented (d, h) strains grown in YPD at 30°C and stained with 0.05% calcofluor white.

The *yck2*Δ/*yck2*Δ strain displaying altered morphology and biofilm likely has cell wall architecture change, which often leads to altered cell wall integrity that in turn may impact cell membrane function. Thus, we examined the susceptibility of the *yck2*Δ/*yck2*Δ strain to various cell wall and cell membrane stressors. As shown in Fig 3A, the *yck2*Δ/*yck2*Δ strain had markedly increased susceptibility to protamine, SDS, congo red, and calcofluor white. In contrast, the *yck2*Δ/*yck2*Δ strain grew normally in the presence of NaCl or H_2_O_2_, indicating that CaYck2p is not required for its resistance to either osmotic or oxidative stress. This result suggests that CaYck2p is involved in governing cell wall integrity but is not related to either osmotic or oxidative stress response.

To further investigate the degree of hypersensitivity of the *yck2*Δ*/yck2*Δ strain to the cell wall stressors, the minimum inhibitory concentration (MIC) values of fluconazole and caspofungin were measured. As shown on Table 1, the 24 h and 48 h fluconazole MICs for the *yck2*Δ*/yck2*Δ strain were 0.062 μg/ml and 0.125 μg/ml, respectively, which were hypersusceptible compared with those for wild type (0.5 μg/ml at 24 h and 1 μg/ml at 48 h) and complemented strains (0.25 μg/ml at 24 h and 0.5 μg/ml at 48 h). The MIC values of caspofungin demonstrated similar results; the MICs for *yck2*Δ*/yck2*Δ strain were significantly lower (0.031 μg/ml at 24 h and 0.125 μg/ml at 48 h) than those for the wild-type strain and the complemented strains (0.5 μg/ml at 24 h and 1 μg/ml at 48 h).

**Table 1:** Minimum inhibitory concentrations of fluconazole and caspofungin for the *yck2*Δ*/yck2*Δ strain.

	Minimum Inhibitory Concentration (μg/mL)			
*C*. *albcans* strains	Fluconazole		Caspofungin	
	24 h	48 h	24 h	48 h
Wild-type (DIC185)	0.5	1	0.5	1
*yck*2Δ/*yck2*Δ (1041U)	0.062	0.125	0.031	0.125
*yck2*Δ/*yck2*Δ + *YCK*2 (10410)	0.5	0.5	0.5	1

We sought to identify potential regulatory mechanisms by which CaYck2p maintains cell wall integrity. To test whether CaYck2p is associated with the Ca^2+^/calcineurin pathway in governing cell wall integrity, the susceptibility of the *yck2*Δ/*yck2*Δ strain to cyclosporine A and FK506 was tested [48]. As shown on Fig 3B, the wild-type strain was resistant to 10 μg/ml cyclosporine A and 5 μg/ml FK506. The *C*. *albicans vps15*Δ*/vps15*Δ strain is known to be hypersusceptible to both inhibitors [37], and, thus, was used as a positive control for susceptibility to these drugs. The *yck2*Δ/*yck2*Δ strain grew similarly to the wild-type strain, suggesting that Yck2p does not influence cell wall integrity via the calcineurin pathway. To test the conserved function of the CK1 family among eukaryotic system, the *yck2*Δ/*yck2*Δ strain was also tested for its susceptibility to pyrvinium pamoate, a drug that modulates the CK1 and Wnt signaling pathways [49, 50]. The *yck2*Δ/*yck2*Δ strain displayed significantly increased sensitivity to pyrvinium pamoate as compared to the wild-type strain (Fig 3B), which provides evidence that CaYck2p is functions in the CK1 and Wnt signaling pathways.

### Compensatory chitin synthesis was induced by the loss of Yck2p function

The finding that the *yck2*Δ/*yck2*Δ strain was sensitive to cell wall targeting drugs suggested that the *yck2*Δ/*yck2*Δ strain may have a compensatory increase in chitin deposition. When the *yck2*Δ/*yck2*Δ strain was stained with calcofluor white to assess chitin levels and distribution in the cell wall, there was notably increased fluorescence in the cell wall and the septum of the *yck2*Δ/*yck2*Δ strain as compared to both the wild-type and the complemented strains (Fig 3C). This result suggests that there is an increased chitin synthesis in the absence of functional CaYck2p.

**Fig 4:**
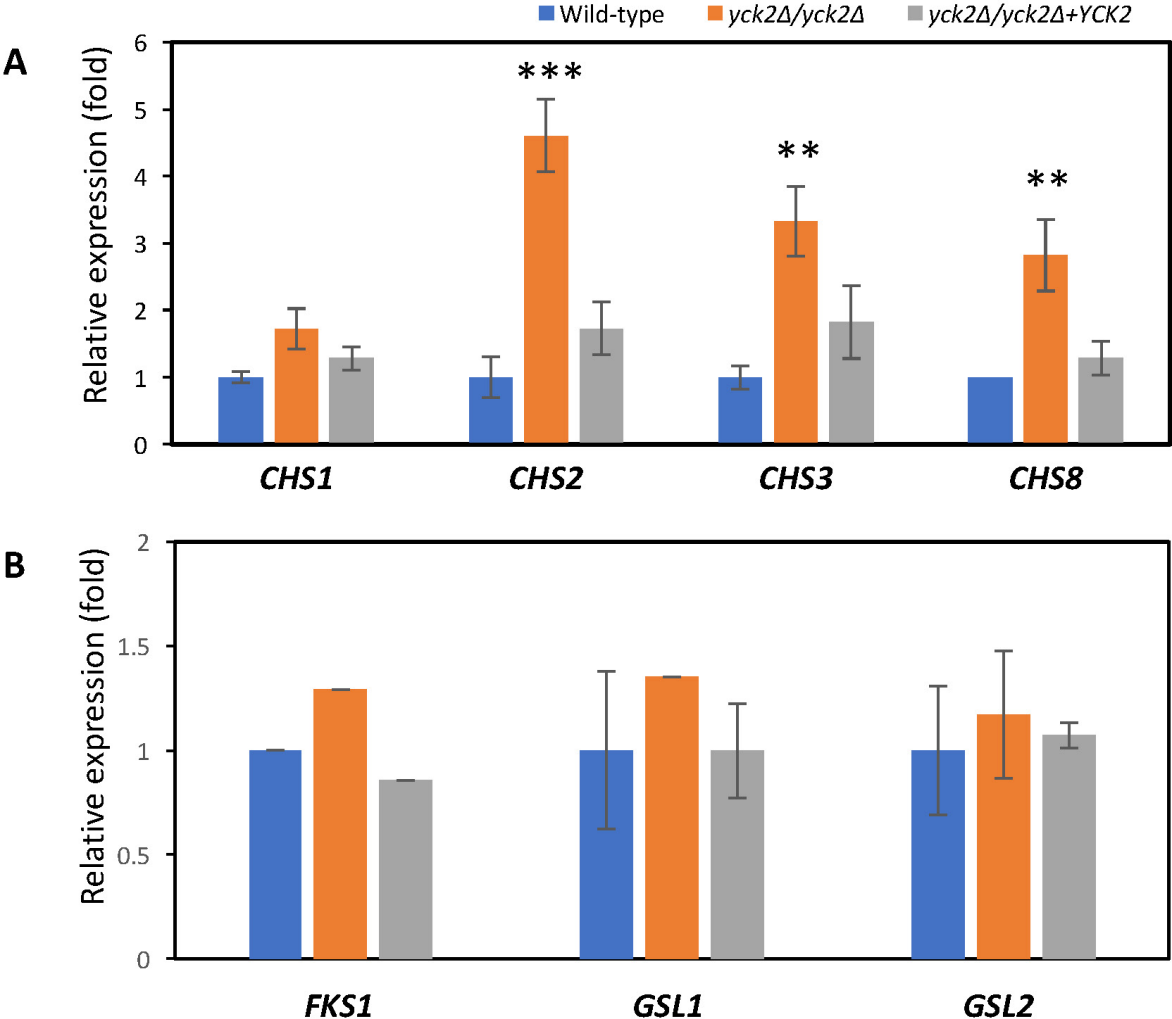
Quantitative gene expression analysis of cell wall synthase genes. **A,** the relative mRNA expression of chitin synthase genes compared to wild type in the indicated strains grown in YPD at 30°C to mid log phase (** p<0.001 and *** p<0.0001). **B,** the relative mRNA expression of glucan synthase genes compared to wild type in the indicated strains grown in YPD at 30°C to mid log phase.

Transcriptional analysis of the chitin synthase genes in the *yck2*Δ*/yck2*Δ strain demonstrated that the expression of *CHS2*, *CHS3*, and *CHS8* genes was induced by 5-fold (p<0.0001), 3.3-fold (p<0.01), and 3-fold (p<0.01), respectively, compared to the wild-type strain and the complemented strains (Fig 4A). *CHS1* expression was slightly, but not significantly increased in the *yck2*Δ*/yck2*Δ strain. These data suggest that the loss of functional CaYck2p results in impaired cell wall integrity, which then leads to a compensatory increase in chitin synthase mRNA expression, leading to enhance cell wall chitin deposition. However, the transcription level of glucan synthase genes *(FKS1*, *GSL1*, *GSL2*) were similar between the *yck2*Δ/*yck2*Δ and the wild-type strains (Fig 4B).

### Yck2p is involved in hyphal specific gene regulation

**Fig 5:**
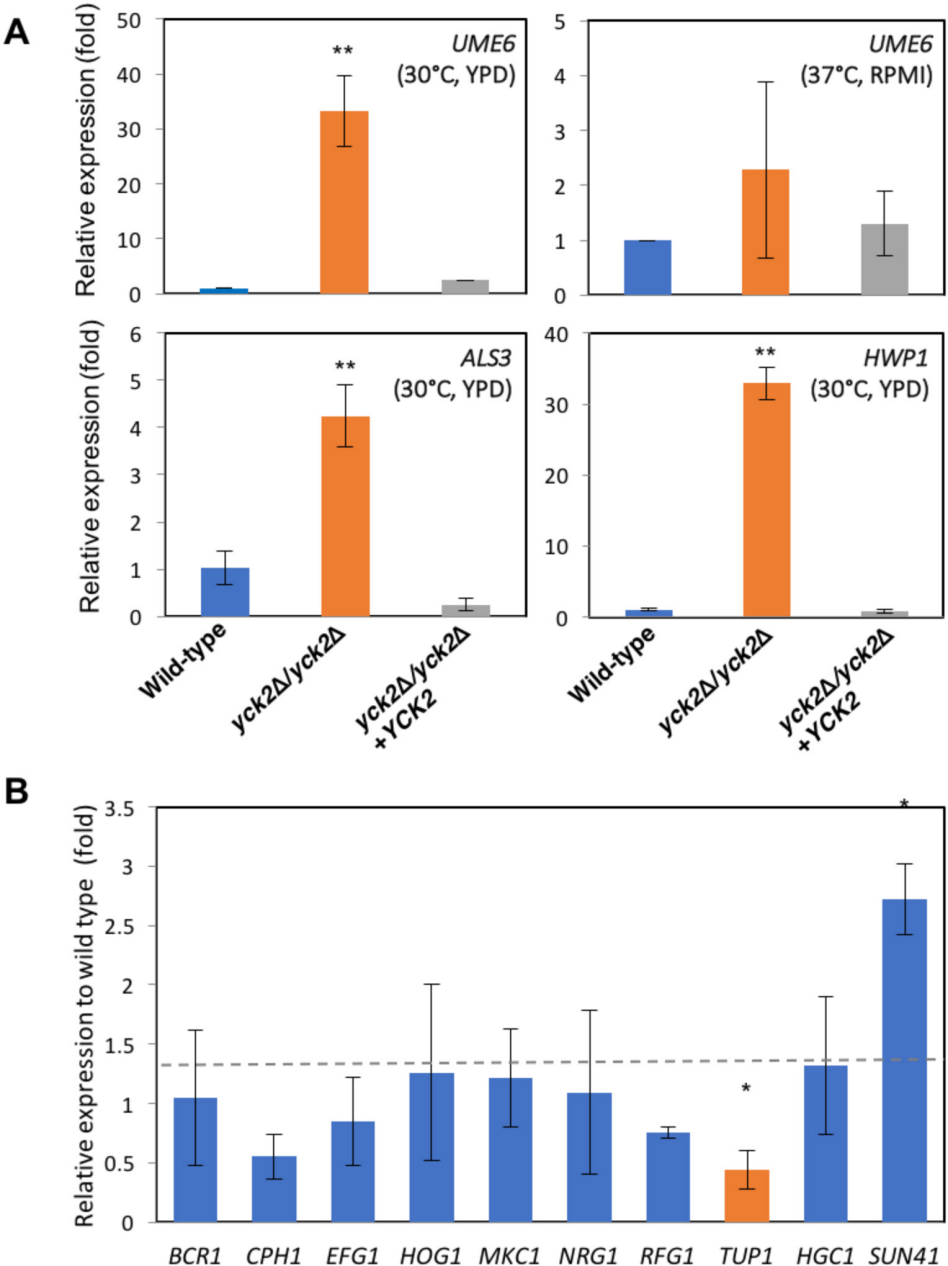
Quantitative gene expression analysis. **A,** relative mRNA expression of *UME6*, *ALS3*, and *HWP1* in the indicated strains grown in YPD at 30°C and in RPMI at 37°C, respectively. Shown are means ± S.D. of three independent experiments (** p<0.001). **B,** relative mRNA expression of genes involved in morphology transition and biofilm formation in the *yck2Δ/yck2Δ* strain compared to the wild type strain grown in YPD at 30°C. Shown are means ± SD for three biological replicates (* p<0.05).

To determine how CaYck2p regulates *C*. *albicans* morphology, biofilm formation, and host cell damage, we assessed the transcription levels of the genes associated with these processes. First, we tested the mRNA expression of a transcription factor, *UME6*, for which has increased expression during the yeast-to-hyphal transition [51]. *UME6* mRNA levels in the *yck2*Δ*/yck2*Δ strain grown yeast-inducing condition (YPD at 30°C) were 35-fold higher than in the wild-type strain (p<0.001) (Fig 5A, top left). The complemented strain expressed wild-type levels of *UME6*. When the strains were grown under hyphal inducing conditions, all strains expressed similar levels of *UME6* (Fig 5A, top right). The increased *UME6* expression in the *yck2*Δ*/yck2*Δ strain is likely the cause of its pseudohyphal growth and increased biofilm formation under yeast-inducing condition. We also measured the transcription of *ALS3* and *HWP1*, both of which are highly expressed by hyphae, and involved in biofilm formation [52]. Fig 5A (bottom) demonstrated that there was a 4-fold increased expression of *ALS3* (p<0.001) and a 32-fold increased level of *HWP1* (p<0.001) relative to the wild-type strain, even when the organisms were grown under yeast-inducing conditions.

To further elucidate the molecular mechanisms of the altered phenotype of the *yck2*Δ*/yck2*Δ strain, we measured the mRNA expression levels of *BCR1*, *CPH1*, and *EFG1*, which encode transcription factors known to be responsible for hyphal transition and/or biofilm formation. *CPH1* expression was slightly decreased but the change was not statistically significant. Either *BCR1* or *EFG1* was not differentially expressed in the *yck2*Δ*/yck2*Δ strain relative to the wild-type, as shown on Fig 5B, suggesting that CaYck2p is not involved in the transcriptional regulation of these genes. The transcription of *HOG1* and *MKC1*, which specify MAP kinases involved in stress response, was not altered in the *yck2*Δ*/yck2*Δ strain as compared to the wild-type strain. The expression of transcription genes encoding the repressor proteins, *NRG1*, *RFG1*, and *TUP1*, which represses *UME6* transcription and hyphal transition was also tested. Only *TUP1* expression was decreased in the *yck2*Δ/*yck2*Δ strain as compared to the wild type. The expression of *SUN41* was increased in the *yck2Δ/yck2Δ* strain as compared to the wild-type strain, which is consistent with previous finding that increased *UME6* expression enhances *SUN41* transcription [23]. Collectively, these results suggest that CaYck2p is associated with de-repression of *UME6* and its downstream hyphal specific genes, *ALS3*, *HWP1*, and *SUN41* when *C*. *albicans* is grown under yeast-inducing conditions.

## Discussion

This study identified a new function of the *C*. *albicans* yeast casein kinase 2 (CaYck2p) in controlling morphogenesis, biofilm formation, host cell damage, and cell wall integrity. Our results suggest that CaYck2p is the dominant CK1 homolog of *C*. *albicans*, by which governs similar biological processes as ScYck1p/ScYck2p [26, 31, 53, 54] of *S*. *cerevisiae* and other fungal CK1 homologs [32, 55, 56]. We noticed that the morphology of the *yck2*Δ/*yck2*Δ strain differed significantly from that of the insertion mutant strain reported in our previous study [37]. The insertion mutant strain, expressing a truncated CaYck2p with the conserved catalytic domain, had delayed yeast-to-hyphal transition [37]. By contrast, the *yck2*Δ/*yck2*Δ strain was not able to switch between yeast and hyphae and formed constitutive pseudohyphae-like cells. Thus, the N-terminus catalytic domain of CaYck2p seems to be crucial for the yeast-to-hyphal transition. The transcription of *YCK3* was increased in the *yck2*Δ/*yck2*Δ strain, suggesting that there was compensatory upregulation of the other CK1 homolog in the absence of CaYck2p. Still, CaYck2p and CaYck3p do not seem to play interchangeable function as the lack of functional CaYck2p alone resulted similar morphological changes, similar to the double ScYck1p/ScYck2p mutations [26, 31, 53, 54].

The *yck2*Δ/*yck2*Δ strain grew as a constitutive pseudohyphae-like form in both yeast and hyphal growth conditions. Yet, the microscopic observation alone did not provide adequate evidence of whether the mutant filaments were bona fide pseudohyphae or defected filamentation under hyphal inducing condition. The capacity of *C*. *albicans* to switch between yeast, pseudohyphae, and hyphae forms contributes significantly to its ability to form biofilm [23, 52, 57]. The *yck2*Δ/*yck2*Δ strain formed a biofilm under various non-biofilm inducing conditions, and formed similar wild-type biofilm under strong biofilm inducing conditions. Thus, it is likely that the constitutive pseudohyphae of the *yck2*Δ/*yck2*Δ strain enabled the organism to form a biofilm even under non-inducing condition. Studies suggest that the pseudohyphae are intermediate to yeast and hyphae and the genes associated with pseudohyphal form are a subset of those with hyphal form [7, 58]. Of those genes, *UME6* is transcriptionally regulated, and its mRNA expression levels escalate as the cell transition from yeast to pseudohyphae to true hyphae. Previous studies also demonstrated that overexpression of *UME6* in a non-biofilm forming mutant restored biofilm formation [20, 59] and significantly increased levels of *ALS3*, which encodes an agglutinin-like protein [60–62] and invasin [63, 64], and *HWP1*, which specifies a hyphal specific protein involved in adherence and biofilm formation [52, 65–67]. We found that the *yck2*Δ/*yck2*Δ strain overexpresses *UME6*, as well as *ALS3*, and *HWP1*. Thus, we speculate that in the *yck2*Δ/*yck2*Δ strain, de-repression of *UME6* led to the increased *ALS3* and *HWP1* to the enhanced biofilm formation.

As the filamentous growth program is initiated by relief of transcriptional repression [68], CaYck2p is likely associated with transcriptional repression of *UME6* and its downstream genes. It was reported that the transcription of *UME6* is upregulated by Efg1p and negatively regulated by Nrg1p-Tup1p and Rfg1p-Tup1p repression complexes [51, 69, 70]. Thus, it is possible that CaYck2p interacts with the transcription factors that regulate *UME6* transcription. Our study found the expression of *TUP1* was slightly decreased in the *yck2*Δ/*yck2*Δ strain. Thus, reduced *TUP1* expression in the *yck2*Δ/*yck2*Δ strain likely decreases the repressive activity the Tup1 complexes, leading to de-repression of *UME6* transcription. Nevertheless, the *yck2*Δ/*yck2*Δ strain is locked in pseudohyphae form even with the elevated *UME6* expression, in contrast to the previous result that constitutive expression of *UME6* induces the formation of true hyphae under non-hyphae inducing condition [7, 23, 51, 71]. As *tup1*Δ*/tup1*Δ mutant is known to grow constitutively as pseudohyphae [4], reduced *TUP1* expression may be one reason that the *yck2*Δ*/yck2*Δ strain has this morphology. Therefore, we conclude that the elevated *UME6* expression is insufficient to induce the formation of true hyphae in the absence of functional CaYck2p. This study also suggests that true hyphal formation is not required for in vitro biofilm formation; the increased *ALS3* and *HWP1* expression in pseudohyphae of the *yck2*Δ/*yck2*Δ strain was sufficient to promote constitutive biofilm formation. Still, the biofilm formed by the *yck2*Δ/*yck2*Δ strain might have different ultrastructure as compared to the wild-type strain. In the future, a closer investigation of the role of Yck2p in governing biofilm architecture and the process of cell dispersal from mature biofilms will provide additional insight into the role of Yck2p in biofilm formation and dispersal.

Many studies suggest that the *C*. *albicans* strains with impaired morphogenesis also have reduced virulence [72, 73]. Our result demonstrated that the *yck2*Δ/*yck2*Δ strain, even with increased expression of *UME6* and its downstream cell surface adhesins, *ALS3* and *HWP1*, had significantly decreased ability to damage both endothelial and epithelial cells tested in this study. These results strongly suggest that the *yck2*Δ/*yck2*Δ strain would have reduced virulence in mouse models of mucosal and disseminated infection.

Cell wall dynamics are controlled by a broad signaling network [74, 75] and are tightly linked to *C*. *albicans* growth, morphogenesis, and pathogenicity [76]. The *yck2*Δ/*yck2*Δ strain was hypersensitive to cell wall stressors and demonstrated increased chitin deposition in the cell wall. Transcriptional analysis confirmed increased expression of three chitin synthase genes, *CHS2*, *CHS3*, and *CHS8*, which is consistent with previous studies showing that fungal cells increase chitin synthesis to overcome compromised cell wall integrity [77–79]. The transcription of *SUN41*, a cell wall glycosidase involved in cell wall biogenesis and biofilm formation [80], was also induced in the *yck2*Δ/*yck2*Δ strain as compared to the wild-type. Taken together, these results indicate that CaYck2p is important to cell wall integrity. These data are also consistent with those of a previous study that found that CaYck2p is a part of a carbohydrate utilization protein kinase cluster involved in cell wall biogenesis [36].

One possible mechanism by which CaYck2p is involved in regulation of cell wall integrity is by interacting with one of the cell wall integrity regulatory pathways [81–84]. Our results indicated that the *yck2*Δ/*yck2*Δ strain displayed wild-type sensitivity to cyclosporine A and FK506, suggesting that the absence of CaYck2p does not activate the calcineurin pathway. The PKC-Mkc1p pathway regulates cell wall integrity by directly increasing chitin synthase gene expression when the cell wall is damaged by cell wall perturbing drugs [36, 85, 86]. As the *yck2*Δ/*yck2*Δ strain had increased chitin deposition on the cell wall and the upregulation of chitin synthase genes, it is likely that aberrant cell wall formation in the *yck2*Δ/*yck2*Δ strain mimics stress conditions and leads to constitutive upregulation of compensatory chitin synthesis. A previous study suggested that Cck1p, the CK1 homolog in *C*. *neoformans*, regulates the expression and phosphorylation of Mpk1p and Hog1p under oxidative and osmotic stress conditions [32]. By contrast, we found that the *yck2*Δ/*yck2*Δ strain displayed wild-type resistance to osmotic and oxidative stress. Thus, it is likely that there is functional divergence of CK1 family among fungal species. Finally, we found that pyrvinium pamoate, an anthelminthic agent known to modulate CK1 and Wnt signaling pathway in higher eukaryotic systems, has specific inhibitory effect on the *yck2*Δ/*yck2*Δ strain. This result indicates that Yck2p functions in the CK1/Wnt signaling pathway and suggests that the combination of pyrvinium pamoate and a CK1 inhibitor could be developed into a new antifungal agent.

In summary, the fungal CK1 homologs have conserved their functions in maintaining cellular processes, but species specific functional divergence has occurred. In *C*. *albicans*, Yck2p regulates a range of cellular functions including morphogenesis, biofilm formation, cell wall integrity, and host cell interaction. In particular, CaYck2p likely plays a significant role in governing genetic repression of hyphal specific genes, and in regulating compensatory cell wall biogenesis.

## Supporting information

S1 Fig Sequence analysis of CaYck2p and construction of the homozygous deletion strain. (A) Protein similarity analysis of C. albicans Yck2p, Yck3p, S. cerevisiae Yck1p, and Yck2p. (B) The protein conserved domain information of the CaYck2p (http://www.ncbi.nlm.nih.gov/Structure/cdd/wrpsb.cgi). (C) Illustration of two-step gene disruption with HIS1 and ARG4 markers. (D) PCR amplified *YCK2* alleles with YCK2 Confirm-5 and YCK2 Confirm-3 primers. Indicated strains’ genomic DNAs were amplified with YCK2 Confirm-5 and YCK2 Confirm-3 primers, run on 0.8% agarose gel with 1 Kb ladder (New England Biolabs), and imaged with UVP Gel Doc-It system (UVP, CA).
(TIFF)

S2 Fig Colony morphology of the *yck2*Δ/*yck2*Δ strain. Colony morphologies of the wild-type (a), the *yck2*Δ/*yck2*Δ (b), and the *yck2*Δ/*yck2*Δ+*YCK2* complemented (c) strains grown on YPD with 10% serum plate at 37°C for 2 days.
(TIFF)

S3 Fig Extent of biofilm formation by the wild-type strain in various conditions. The extent of biofilm formation in RPMI1640, spider, YPD and YPglycerol media at 30°C and 37°C was tested by colormetric absorbance analysis at 595nm. (** p<0.01 in OneWay ANOVA with Dunnett’s test posthoc analysis).
(TIFF)

S1 Table Strains used in this study. (PDF)

S2 Table Primers used in this study. (PDF)

## References

[1] Pfaller MA, Diekema DJ. Epidemiology of invasive mycoses in North America. Crit Rev Microbiol. 2010;36(1):1–53. doi: 10.3109/10408410903241444 . 20088682 10.3109/10408410903241444

[2] Arendrup MC. Epidemiology of invasive candidiasis. Curr Opin Crit Care. 2010;16(5):445–52. doi: 10.1097/MCC.0b013e32833e84d2 . 20711075 10.1097/MCC.0b013e32833e84d2

[3] Lo HJ, Kohler JR, DiDomenico B, Loebenberg D, Cacciapuoti A, Fink GR. Nonfilamentous *C*. *albicans* mutants are avirulent. Cell. 1997;90(5):939–49. . 9298905 10.1016/s0092-8674(00)80358-x

[4] Braun BR, Johnson AD. Control of filament formation in *Candida albicans* by the transcriptional repressor TUP1. Science. 1997;277(5322):105–9. . 9204892 10.1126/science.277.5322.105

[5] Braun BR, Johnson AD. TUP1, CPH1 and EFG1 make independent contributions to filamentation in *Candida albicans*. Genetics. 2000;155(1):57–67. ; PubMed Central PMCID: PMCPMC1461068. 10790384 10.1093/genetics/155.1.57 PMC1461068

[6] Kumamoto CA, Vinces MD. Contributions of hyphae and hypha-co-regulated genes to *Candida albicans* virulence. Cell Microbiol. 2005;7(11):1546–54. doi: 10.1111/j.1462-5822.2005.00616.x . 16207242 10.1111/j.1462-5822.2005.00616.x

[7] Carlisle PL, Banerjee M, Lazzell A, Monteagudo C, Lopez-Ribot JL, Kadosh D. Expression levels of a filament-specific transcriptional regulator are sufficient to determine *Candida albicans* morphology and virulence. Proc Natl Acad Sci U S A. 2009;106(2):599–604. doi: 10.1073/pnas.0804061106 ; PubMed Central PMCID: PMCPMC2626749. 19116272 10.1073/pnas.0804061106 PMC2626749

[8] Gow NA, Brown AJ, Odds FC. Fungal morphogenesis and host invasion. Curr Opin Microbiol. 2002;5(4):366–71. . 12160854 10.1016/s1369-5274(02)00338-7

[9] Brown AJ, Gow NA. Regulatory networks controlling *Candida albicans* morphogenesis. Trends Microbiol. 1999;7(8):333–8. . 10431207 10.1016/s0966-842x(99)01556-5

[10] Park H, Myers CL, Sheppard DC, Phan QT, Sanchez AA, E Edwards J, et al Role of the fungal Ras‐protein kinase A pathway in governing epithelial cell interactions during oropharyngeal candidiasis. Cellular microbiology. 2005;7(4):499–510. doi: 10.1111/j.1462-5822.2004.00476.x 15760450 10.1111/j.1462-5822.2004.00476.x

[11] Gow NA, van de Veerdonk FL, Brown AJ, Netea MG. *Candida albicans* morphogenesis and host defence: discriminating invasion from colonization. Nat Rev Microbiol. 2012;10(2):112–22. doi: 10.1038/nrmicro2711 ; PubMed Central PMCID: PMCPMC3624162. 22158429 10.1038/nrmicro2711 PMC3624162

[12] Inglis DO, Sherlock G. Ras signaling gets fine-tuned: regulation of multiple pathogenic traits of *Candida albicans*. Eukaryot Cell. 2013;12(10):1316–25. doi: 10.1128/EC.00094-13 ; PubMed Central PMCID: PMCPMC3811338. 23913542 10.1128/EC.00094-13 PMC3811338

[13] Saputo S, Kumar A, Krysan DJ. Efg1 directly regulates ACE2 expression to mediate cross talk between the cAMP/PKA and RAM pathways during *Candida albicans* morphogenesis. Eukaryot Cell. 2014;13(9):1169–80. doi: 10.1128/EC.00148-14 ; PubMed Central PMCID: PMCPMC4187626. 25001410 10.1128/EC.00148-14 PMC4187626

[14] Sudbery PE. Growth of *Candida albicans* hyphae. Nat Rev Microbiol. 2011;9(10):737–48. doi: 10.1038/nrmicro2636 . 21844880 10.1038/nrmicro2636

[15] Nobile CJ, Mitchell AP. Regulation of cell-surface genes and biofilm formation by the *C*. *albicans* transcription factor Bcr1p. Curr Biol. 2005;15(12):1150–5. doi: 10.1016/j.cub.2005.05.047 . 15964282 10.1016/j.cub.2005.05.047

[16] Ramage G, Saville SP, Thomas DP, Lopez-Ribot JL. *Candida* biofilms: an update. Eukaryot Cell. 2005;4(4):633–8. doi: 10.1128/EC.4.4.633-638.2005 ; PubMed Central PMCID: PMCPMC1087806. 15821123 10.1128/EC.4.4.633-638.2005 PMC1087806

[17] Richard ML, Nobile CJ, Bruno VM, Mitchell AP. *Candida albicans* biofilm-defective mutants. Eukaryot Cell. 2005;4(8):1493–502. doi: 10.1128/EC.4.8.1493-1502.2005 ; PubMed Central PMCID: PMCPMC1214533. 16087754 10.1128/EC.4.8.1493-1502.2005 PMC1214533

[18] Fanning S, Xu W, Beaurepaire C, Suhan JP, Nantel A, Mitchell AP. Functional control of the *Candida albicans* cell wall by catalytic protein kinase A subunit Tpk1. Mol Microbiol. 2012;86(2):284–302. doi: 10.1111/j.1365-2958.2012.08193.x ; PubMed Central PMCID: PMCPMC3947901. 22882910 10.1111/j.1365-2958.2012.08193.x PMC3947901

[19] Chandra J, Kuhn DM, Mukherjee PK, Hoyer LL, McCormick T, Ghannoum MA. Biofilm formation by the fungal pathogen *Candida albicans*: development, architecture, and drug resistance. J Bacteriol. 2001;183(18):5385–94. doi: 10.1128/JB.183.18.5385-5394.2001 ; PubMed Central PMCID: PMCPMC95423. 11514524 10.1128/JB.183.18.5385-5394.2001 PMC95423

[20] Desai JV, Bruno VM, Ganguly S, Stamper RJ, Mitchell KF, Solis N, et al Regulatory role of glycerol in *Candida albicans* biofilm formation. MBio. 2013;4(2):e00637–12. doi: 10.1128/mBio.00637-12 ; PubMed Central PMCID: PMCPMC3622937. 23572557 10.1128/mBio.00637-12 PMC3622937

[21] Mukherjee PK, Mohamed S, Chandra J, Kuhn D, Liu S, Antar OS, et al Alcohol dehydrogenase restricts the ability of the pathogen *Candida albicans* to form a biofilm on catheter surfaces through an ethanol-based mechanism. Infect Immun. 2006;74(7):3804–16. doi: 10.1128/IAI.00161-06 ; PubMed Central PMCID: PMCPMC1489753. 16790752 10.1128/IAI.00161-06 PMC1489753

[22] Finkel JS, Mitchell AP. Genetic control of *Candida albicans* biofilm development. Nat Rev Microbiol. 2011;9(2):109–18. doi: 10.1038/nrmicro2475 ; PubMed Central PMCID: PMCPMC3891587. 21189476 10.1038/nrmicro2475 PMC3891587

[23] Banerjee M, Uppuluri P, Zhao XR, Carlisle PL, Vipulanandan G, Villar CC, et al Expression of UME6, a key regulator of *Candida albicans* hyphal development, enhances biofilm formation via Hgc1- and Sun41-dependent mechanisms. Eukaryot Cell. 2013;12(2):224–32. doi: 10.1128/EC.00163-12 ; PubMed Central PMCID: PMCPMC3571304. 23223035 10.1128/EC.00163-12 PMC3571304

[24] Blankenship JR, Mitchell AP. How to build a biofilm: a fungal perspective. Curr Opin Microbiol. 2006;9(6):588–94. doi: 10.1016/j.mib.2006.10.003 . 17055772 10.1016/j.mib.2006.10.003

[25] Gow NA, Hube B. Importance of the *Candida albicans* cell wall during commensalism and infection. Curr Opin Microbiol. 2012;15(4):406–12. doi: 10.1016/j.mib.2012.04.005 . 22609181 10.1016/j.mib.2012.04.005

[26] Robinson LC, Hubbard EJ, Graves PR, DePaoli-Roach AA, Roach PJ, Kung C, et al Yeast casein kinase I homologues: an essential gene pair. Proc Natl Acad Sci U S A. 1992;89(1):28–32. ; PubMed Central PMCID: PMCPMC48168. 1729698 10.1073/pnas.89.1.28 PMC48168

[27] Davidson G. The cell cycle and Wnt. Cell Cycle. 2010;9(9):1667–8. doi: 10.4161/cc.9.9.11595 . 20404508 10.4161/cc.9.9.11595

[28] Cheong JK, Virshup DM. Casein kinase 1: Complexity in the family. Int J Biochem Cell Biol. 2011;43(4):465–9. doi: 10.1016/j.biocel.2010.12.004 . 21145983 10.1016/j.biocel.2010.12.004

[29] Panek HR, Stepp JD, Engle HM, Marks KM, Tan PK, Lemmon SK, et al Suppressors of YCK-encoded yeast casein kinase 1 deficiency define the four subunits of a novel clathrin AP-like complex. EMBO J. 1997;16(14):4194–204. ; PubMed Central PMCID: PMCPMC1170045. 9250663 10.1093/emboj/16.14.4194 PMC1170045

[30] Kearney PH, Ebert M, Kuret J. Molecular cloning and sequence analysis of two novel fission yeast casein kinase-1 isoforms. Biochem Biophys Res Commun. 1994;203(1):231–6. doi: 10.1006/bbrc.1994.2172 . 8074660 10.1006/bbrc.1994.2172

[31] Robinson LC, Menold MM, Garrett S, Culbertson MR. Casein kinase I-like protein kinases encoded by YCK1 and YCK2 are required for yeast morphogenesis. Mol Cell Biol. 1993;13(5):2870–81. ; PubMed Central PMCID: PMCPMC359678. 8474447 10.1128/mcb.13.5.2870-2881.1993 PMC359678

[32] Wang Y, Liu TB, Patel S, Jiang L, Xue C. The casein kinase I protein Cck1 regulates multiple signaling pathways and is essential for cell integrity and fungal virulence in *Cryptococcus neoformans*. Eukaryot Cell. 2011;10(11):1455–64. doi: 10.1128/EC.05207-11 ; PubMed Central PMCID: PMCPMC3209051. 21926330 10.1128/EC.05207-11 PMC3209051

[33] Robinson LC, Bradley C, Bryan JD, Jerome A, Kweon Y, Panek HR. The Yck2 yeast casein kinase 1 isoform shows cell cycle-specific localization to sites of polarized growth and is required for proper septin organization. Mol Biol Cell. 1999;10(4):1077–92. ; PubMed Central PMCID: PMCPMC25234. 10198058 10.1091/mbc.10.4.1077 PMC25234

[34] Moriya H, Johnston M. Glucose sensing and signaling in *Saccharomyces cerevisiae* through the Rgt2 glucose sensor and casein kinase I. Proc Natl Acad Sci U S A. 2004;101(6):1572–7. doi: 10.1073/pnas.0305901101 ; PubMed Central PMCID: PMCPMC341776. 14755054 10.1073/pnas.0305901101 PMC341776

[35] Brown V, Sabina J, Johnston M. Specialized sugar sensing in diverse fungi. Curr Biol. 2009;19(5):436–41. doi: 10.1016/j.cub.2009.01.056 ; PubMed Central PMCID: PMCPMC2762733. 19249212 10.1016/j.cub.2009.01.056 PMC2762733

[36] Blankenship JR, Fanning S, Hamaker JJ, Mitchell AP. An extensive circuitry for cell wall regulation in *Candida albicans*. PLoS Pathog. 2010;6(2):e1000752 doi: 10.1371/journal.ppat.1000752 ; PubMed Central PMCID: PMCPMC2816693. 20140194 10.1371/journal.ppat.1000752 PMC2816693

[37] Park H, Liu Y, Solis N, Spotkov J, Hamaker J, Blankenship JR, et al Transcriptional responses of *Candida albicans* to epithelial and endothelial cells. Eukaryotic cell. 2009;8(10):1498–510. doi: 10.1128/EC.00165-09 19700637 10.1128/EC.00165-09 PMC2756863

[38] Wilson RB, Davis D, Mitchell AP. Rapid hypothesis testing with *Candida albicans* through gene disruption with short homology regions. J Bacteriol. 1999;181(6):1868–74. ; PubMed Central PMCID: PMCPMC93587. 10074081 10.1128/jb.181.6.1868-1874.1999 PMC93587

[39] Phan QT, Eng DK, Mostowy S, Park H, Cossart P, Filler SG. Role of endothelial cell septin 7 in the endocytosis of *Candida albicans*. MBio. 2013;4(6):e00542–13. doi: 10.1128/mBio.00542-13 24345743 10.1128/mBio.00542-13 PMC3870263

[40] Jin Y, Yip HK, Samaranayake YH, Yau JY, Samaranayake LP. Biofilm-forming ability of *Candida albicans* is unlikely to contribute to high levels of oral yeast carriage in cases of human immunodeficiency virus infection. J Clin Microbiol. 2003;41(7):2961–7. doi: 10.1128/JCM.41.7.2961-2967.2003 ; PubMed Central PMCID: PMCPMC165379. 12843027 10.1128/JCM.41.7.2961-2967.2003 PMC165379

[41] Park H, Liu Y, Solis N, Spotkov J, Hamaker J, Blankenship JR, et al Transcriptional responses of *Candida albicans* to epithelial and endothelial cells. Eukaryot Cell. 2009;8(10):1498–510. doi: 10.1128/EC.00165-09 ; PubMed Central PMCID: PMCPMC2756863. 19700637 10.1128/EC.00165-09 PMC2756863

[42] Espinel-Ingroff A, Canton E, Peman J, Rinaldi MG, Fothergill AW. Comparison of 24-hour and 48-hour voriconazole MICs as determined by the Clinical and Laboratory Standards Institute broth microdilution method (M27-A3 document) in three laboratories: results obtained with 2,162 clinical isolates of Candida spp. and other yeasts. J Clin Microbiol. 2009;47(9):2766–71. doi: 10.1128/JCM.00654-09 ; PubMed Central PMCID: PMCPMC2738097. 19571029 10.1128/JCM.00654-09 PMC2738097

[43] Jaffe EA, Nachman RL, Becker CG, Minick CR. Culture of human endothelial cells derived from umbilical veins. Identification by morphologic and immunologic criteria. J Clin Invest. 1973;52(11):2745–56. doi: 10.1172/JCI107470 ; PubMed Central PMCID: PMCPMC302542. 4355998 10.1172/JCI107470 PMC302542

[44] Dickson MA, Hahn WC, Ino Y, Ronfard V, Wu JY, Weinberg RA, et al Human keratinocytes that express hTERT and also bypass a p16(INK4a)-enforced mechanism that limits life span become immortal yet retain normal growth and differentiation characteristics. Mol Cell Biol. 2000;20(4):1436–47. ; PubMed Central PMCID: PMCPMC85304. 10648628 10.1128/mcb.20.4.1436-1447.2000 PMC85304

[45] Fichorova RN, Rheinwald JG, Anderson DJ. Generation of papillomavirus-immortalized cell lines from normal human ectocervical, endocervical, and vaginal epithelium that maintain expression of tissue-specific differentiation proteins. Biol Reprod. 1997;57(4):847–55. . 9314589 10.1095/biolreprod57.4.847

[46] Filler SG, Swerdloff JN, Hobbs C, Luckett PM. Penetration and damage of endothelial cells by *Candida albicans*. Infect Immun. 1995;63(3):976–83. ; PubMed Central PMCID: PMCPMC173098. 7868270 10.1128/iai.63.3.976-983.1995 PMC173098

[47] Livak KJ, Schmittgen TD. Analysis of relative gene expression data using real-time quantitative PCR and the 2(-Delta Delta C(T)) Method. Methods. 2001;25(4):402–8. doi: 10.1006/meth.2001.1262 . 11846609 10.1006/meth.2001.1262

[48] Ho S, Clipstone N, Timmermann L, Northrop J, Graef I, Fiorentino D, et al The mechanism of action of cyclosporin A and FK506. Clin Immunol Immunopathol. 1996;80(3 Pt 2):S40–5. . 8811062 10.1006/clin.1996.0140

[49] Saraswati S, Alfaro MP, Thorne CA, Atkinson J, Lee E, Young PP. Pyrvinium, a potent small molecule Wnt inhibitor, promotes wound repair and post-MI cardiac remodeling. PLoS One. 2010;5(11):e15521 doi: 10.1371/journal.pone.0015521 ; PubMed Central PMCID: PMCPMC2993965. 21170416 10.1371/journal.pone.0015521 PMC2993965

[50] Xu L, Zhang L, Hu C, Liang S, Fei X, Yan N, et al WNT pathway inhibitor pyrvinium pamoate inhibits the self-renewal and metastasis of breast cancer stem cells. Int J Oncol. 2016;48(3):1175–86. doi: 10.3892/ijo.2016.3337 . 26781188 10.3892/ijo.2016.3337

[51] Banerjee M, Thompson DS, Lazzell A, Carlisle PL, Pierce C, Monteagudo C, et al UME6, a novel filament-specific regulator of *Candida albicans* hyphal extension and virulence. Mol Biol Cell. 2008;19(4):1354–65. doi: 10.1091/mbc.E07-11-1110 ; PubMed Central PMCID: PMCPMC2291399. 18216277 10.1091/mbc.E07-11-1110 PMC2291399

[52] Nobile CJ, Nett JE, Andes DR, Mitchell AP. Function of *Candida albicans* adhesin Hwp1 in biofilm formation. Eukaryot Cell. 2006;5(10):1604–10. doi: 10.1128/EC.00194-06 ; PubMed Central PMCID: PMCPMC1595337. 17030992 10.1128/EC.00194-06 PMC1595337

[53] Wang PC, Vancura A, Mitcheson TG, Kuret J. Two genes in Saccharomyces cerevisiae encode a membrane-bound form of casein kinase-1. Mol Biol Cell. 1992;3(3):275–86. ; PubMed Central PMCID: PMCPMC275529. 1627830 10.1091/mbc.3.3.275 PMC275529

[54] Babu P, Bryan JD, Panek HR, Jordan SL, Forbrich BM, Kelley SC, et al Plasma membrane localization of the Yck2p yeast casein kinase 1 isoform requires the C-terminal extension and secretory pathway function. J Cell Sci. 2002;115(Pt 24):4957–68. . 12432082 10.1242/jcs.00203

[55] Shimada M, Yamamoto A, Murakami-Tonami Y, Nakanishi M, Yoshida T, Aiba H, et al Casein kinase II is required for the spindle assembly checkpoint by regulating Mad2p in fission yeast. Biochem Biophys Res Commun. 2009;388(3):529–32. doi: 10.1016/j.bbrc.2009.08.030 . 19666000 10.1016/j.bbrc.2009.08.030

[56] Koyano T, Barnouin K, Snijders AP, Kume K, Hirata D, Toda T. Casein kinase 1gamma acts as a molecular switch for cell polarization through phosphorylation of the polarity factor Tea1 in fission yeast. Genes Cells. 2015;20(12):1046–58. doi: 10.1111/gtc.12309 ; PubMed Central PMCID: PMCPMC4737401. 26525038 10.1111/gtc.12309 PMC4737401

[57] Desai JV, Mitchell AP. *Candida albicans* Biofilm Development and Its Genetic Control. Microbiol Spectr. 2015;3(3). doi: 10.1128/microbiolspec.MB-0005-2014 ; PubMed Central PMCID: PMCPMC4507287. 26185083 10.1128/microbiolspec.MB-0005-2014 PMC4507287

[58] Carlisle PL, Kadosh D. A genome-wide transcriptional analysis of morphology determination in *Candida albicans*. Mol Biol Cell. 2013;24(3):246–60. doi: 10.1091/mbc.E12-01-0065 ; PubMed Central PMCID: PMCPMC3564527. 23242994 10.1091/mbc.E12-01-0065 PMC3564527

[59] Desai JV, Cheng S, Ying T, Nguyen MH, Clancy CJ, Lanni F, et al Coordination of *Candida albicans* Invasion and Infection Functions by Phosphoglycerol Phosphatase Rhr2. Pathogens. 2015;4(3):573–89. doi: 10.3390/pathogens4030573 ; PubMed Central PMCID: PMCPMC4584273. 26213976 10.3390/pathogens4030573 PMC4584273

[60] Zhao X, Oh SH, Cheng G, Green CB, Nuessen JA, Yeater K, et al ALS3 and ALS8 represent a single locus that encodes a *Candida albicans* adhesin; functional comparisons between Als3p and Als1p. Microbiology. 2004;150(Pt 7):2415–28. doi: 10.1099/mic.0.26943-0 . 15256583 10.1099/mic.0.26943-0

[61] Argimon S, Wishart JA, Leng R, Macaskill S, Mavor A, Alexandris T, et al Developmental regulation of an adhesin gene during cellular morphogenesis in the fungal pathogen *Candida albicans*. Eukaryot Cell. 2007;6(4):682–92. doi: 10.1128/EC.00340-06 ; PubMed Central PMCID: PMCPMC1865654. 17277173 10.1128/EC.00340-06 PMC1865654

[62] Liu Y, Filler SG. *Candida albicans* Als3, a multifunctional adhesin and invasin. Eukaryot Cell. 2011;10(2):168–73. doi: 10.1128/EC.00279-10 ; PubMed Central PMCID: PMCPMC3067396. 21115738 10.1128/EC.00279-10 PMC3067396

[63] Phan QT, Myers CL, Fu Y, Sheppard DC, Yeaman MR, Welch WH, et al Als3 is a *Candida albicans* invasin that binds to cadherins and induces endocytosis by host cells. PLoS Biol. 2007;5(3):e64 doi: 10.1371/journal.pbio.0050064 ; PubMed Central PMCID: PMCPMC1802757. 17311474 10.1371/journal.pbio.0050064 PMC1802757

[64] Fu Y, Phan QT, Luo G, Solis NV, Liu Y, Cormack BP, et al Investigation of the function of *Candida albicans* Als3 by heterologous expression in Candida glabrata. Infect Immun. 2013;81(7):2528–35. doi: 10.1128/IAI.00013-13 ; PubMed Central PMCID: PMCPMC3697595. 23630968 10.1128/IAI.00013-13 PMC3697595

[65] Staab JF, Bradway SD, Fidel PL, Sundstrom P. Adhesive and mammalian transglutaminase substrate properties of *Candida albicans* Hwp1. Science. 1999;283(5407):1535–8. . 10066176 10.1126/science.283.5407.1535

[66] Dwivedi P, Thompson A, Xie Z, Kashleva H, Ganguly S, Mitchell AP, et al Role of Bcr1-activated genes Hwp1 and Hyr1 in *Candida albicans* oral mucosal biofilms and neutrophil evasion. PLoS One. 2011;6(1):e16218 doi: 10.1371/journal.pone.0016218 ; PubMed Central PMCID: PMCPMC3026825. 21283544 10.1371/journal.pone.0016218 PMC3026825

[67] Fan Y, He H, Dong Y, Pan H. Hyphae-specific genes HGC1, ALS3, HWP1, and ECE1 and relevant signaling pathways in *Candida albicans*. Mycopathologia. 2013;176(5–6):329–35. doi: 10.1007/s11046-013-9684-6 . 24002103 10.1007/s11046-013-9684-6

[68] Kadosh D, Johnson AD. Induction of the *Candida albicans* filamentous growth program by relief of transcriptional repression: a genome-wide analysis. Mol Biol Cell. 2005;16(6):2903–12. doi: 10.1091/mbc.E05-01-0073 ; PubMed Central PMCID: PMCPMC1142434. 15814840 10.1091/mbc.E05-01-0073 PMC1142434

[69] Childers DS, Kadosh D. Filament condition-specific response elements control the expression of NRG1 and UME6, key transcriptional regulators of morphology and virulence in *Candida albicans*. PLoS One. 2015;10(3):e0122775 doi: 10.1371/journal.pone.0122775 ; PubMed Central PMCID: PMCPMC4374957. 25811669 10.1371/journal.pone.0122775 PMC4374957

[70] Zeidler U, Lettner T, Lassnig C, Muller M, Lajko R, Hintner H, et al UME6 is a crucial downstream target of other transcriptional regulators of true hyphal development in *Candida albicans*. FEMS Yeast Res. 2009;9(1):126–42. doi: 10.1111/j.1567-1364.2008.00459.x . 19054126 10.1111/j.1567-1364.2008.00459.x

[71] Carlisle PL, Kadosh D. *Candida albicans* Ume6, a filament-specific transcriptional regulator, directs hyphal growth via a pathway involving Hgc1 cyclin-related protein. Eukaryot Cell. 2010;9(9):1320–8. doi: 10.1128/EC.00046-10 ; PubMed Central PMCID: PMCPMC2937344. 20656912 10.1128/EC.00046-10 PMC2937344

[72] Sanchez AA, Johnston DA, Myers C, Edwards JE Jr., Mitchell AP, Filler SG. Relationship between *Candida albicans* virulence during experimental hematogenously disseminated infection and endothelial cell damage in vitro. Infect Immun. 2004;72(1):598–601. doi: 10.1128/IAI.72.1.598-601.2004 ; PubMed Central PMCID: PMCPMC344013. 14688143 10.1128/IAI.72.1.598-601.2004 PMC344013

[73] Phan QT, Belanger PH, Filler SG. Role of hyphal formation in interactions of *Candida albicans* with endothelial cells. Infect Immun. 2000;68(6):3485–90. ; PubMed Central PMCID: PMCPMC97632. 10816502 10.1128/iai.68.6.3485-3490.2000 PMC97632

[74] Bruno VM, Kalachikov S, Subaran R, Nobile CJ, Kyratsous C, Mitchell AP. Control of the *C*. *albicans* cell wall damage response by transcriptional regulator Cas5. PLoS Pathog. 2006;2(3):e21 doi: 10.1371/journal.ppat.0020021 ; PubMed Central PMCID: PMCPMC1401495. 16552442 10.1371/journal.ppat.0020021 PMC1401495

[75] Rauceo JM, Blankenship JR, Fanning S, Hamaker JJ, Deneault JS, Smith FJ, et al Regulation of the *Candida albicans* cell wall damage response by transcription factor Sko1 and PAS kinase Psk1. Mol Biol Cell. 2008;19(7):2741–51. doi: 10.1091/mbc.E08-02-0191 ; PubMed Central PMCID: PMCPMC2441657. 18434592 10.1091/mbc.E08-02-0191 PMC2441657

[76] Chaffin WL, Lopez-Ribot JL, Casanova M, Gozalbo D, Martinez JP. Cell wall and secreted proteins of *Candida albicans*: identification, function, and expression. Microbiol Mol Biol Rev. 1998;62(1):130–80. ; PubMed Central PMCID: PMCPMC98909. 9529890 10.1128/mmbr.62.1.130-180.1998 PMC98909

[77] Walker LA, Munro CA, de Bruijn I, Lenardon MD, McKinnon A, Gow NA. Stimulation of chitin synthesis rescues *Candida albicans* from echinocandins. PLoS Pathog. 2008;4(4):e1000040 doi: 10.1371/journal.ppat.1000040 ; PubMed Central PMCID: PMCPMC2271054. 18389063 10.1371/journal.ppat.1000040 PMC2271054

[78] Walker LA, Lenardon MD, Preechasuth K, Munro CA, Gow NA. Cell wall stress induces alternative fungal cytokinesis and septation strategies. J Cell Sci. 2013;126(Pt 12):2668–77. doi: 10.1242/jcs.118885 ; PubMed Central PMCID: PMCPMC3687699. 23606739 10.1242/jcs.118885 PMC3687699

[79] Stevens DA, Ichinomiya M, Koshi Y, Horiuchi H. Escape of *Candida* from caspofungin inhibition at concentrations above the MIC (paradoxical effect) accomplished by increased cell wall chitin; evidence for beta-1,6-glucan synthesis inhibition by caspofungin. Antimicrob Agents Chemother. 2006;50(9):3160–1. doi: 10.1128/AAC.00563-06 ; PubMed Central PMCID: PMCPMC1563524. 16940118 10.1128/AAC.00563-06 PMC1563524

[80] Hiller E, Heine S, Brunner H, Rupp S. Candida albicans Sun41p, a putative glycosidase, is involved in morphogenesis, cell wall biogenesis, and biofilm formation. Eukaryot Cell. 2007;6(11):2056–65. doi: 10.1128/EC.00285-07 ; PubMed Central PMCID: PMCPMC2168408. 17905924 10.1128/EC.00285-07 PMC2168408

[81] Lagorce A, Hauser NC, Labourdette D, Rodriguez C, Martin-Yken H, Arroyo J, et al Genome-wide analysis of the response to cell wall mutations in the yeast Saccharomyces cerevisiae. J Biol Chem. 2003;278(22):20345–57. doi: 10.1074/jbc.M211604200 . 12644457 10.1074/jbc.M211604200

[82] Boorsma A, de Nobel H, ter Riet B, Bargmann B, Brul S, Hellingwerf KJ, et al Characterization of the transcriptional response to cell wall stress in *Saccharomyces cerevisiae*. Yeast. 2004;21(5):413–27. doi: 10.1002/yea.1109 . 15116342 10.1002/yea.1109

[83] Eisman B, Alonso-Monge R, Roman E, Arana D, Nombela C, Pla J. The Cek1 and Hog1 mitogen-activated protein kinases play complementary roles in cell wall biogenesis and chlamydospore formation in the fungal pathogen *Candida albicans*. Eukaryot Cell. 2006;5(2):347–58. doi: 10.1128/EC.5.2.347-358.2006 ; PubMed Central PMCID: PMCPMC1405885. 16467475 10.1128/EC.5.2.347-358.2006 PMC1405885

[84] Munro CA, Selvaggini S, de Bruijn I, Walker L, Lenardon MD, Gerssen B, et al The PKC, HOG and Ca2+ signalling pathways co-ordinately regulate chitin synthesis in *Candida albicans*. Mol Microbiol. 2007;63(5):1399–413. doi: 10.1111/j.1365-2958.2007.05588.x ; PubMed Central PMCID: PMCPMC2649417. 17302816 10.1111/j.1365-2958.2007.05588.x PMC2649417

[85] Navarro-Garcia F, Sanchez M, Pla J, Nombela C. Functional characterization of the MKC1 gene of *Candida albicans*, which encodes a mitogen-activated protein kinase homolog related to cell integrity. Mol Cell Biol. 1995;15(4):2197–206. ; PubMed Central PMCID: PMCPMC230448. 7891715 10.1128/mcb.15.4.2197 PMC230448

[86] Valdivia RH, Schekman R. The yeasts Rho1p and Pkc1p regulate the transport of chitin synthase III (Chs3p) from internal stores to the plasma membrane. Proc Natl Acad Sci U S A. 2003;100(18):10287–92. doi: 10.1073/pnas.1834246100 ; PubMed Central PMCID: PMCPMC193553. 12928491 10.1073/pnas.1834246100 PMC193553

