# Supplementary figures and images for: Yeast casein kinase 2 governs morphology, biofilm formation, cell wall integrity, and host cell damage of Candida albicans
Source: PLoS One. 2017 Nov 6;12(11):e0187721. doi: 10.1371/journal.pone.0187721 (PMC5673188; doi:10.1371/journal.pone.0187721)

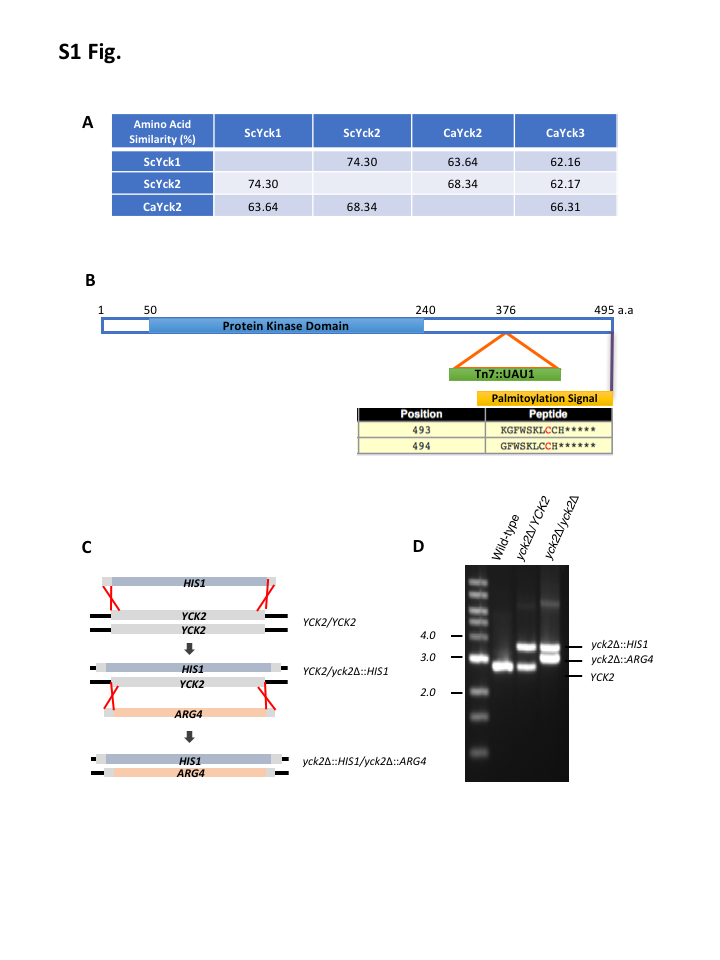

Supplement: S1 Fig — (A) Protein similarity analysis of C. albicans Yck2p, Yck3p, S. cerevisiae Yck1p, and Yck2p. (B) The protein conserved domain information of the CaYck2p (http://www.ncbi.nlm.nih.gov/Structure/cdd/wrpsb.cgi). (C) Illustration of two-step gene disruption with HIS1 and ARG4 markers. (D) PCR amplified YCK2 alleles with YCK2 Confirm-5 and YCK2 Confirm-3 primers. Indicated strains’ genomic DNAs were amplified with YCK2 Confirm-5 and YCK2 Confirm-3 primers, run on 0.8% agarose gel with 1 Kb ladder (New England Biolabs), and imaged with UVP Gel Doc-It system (UVP, CA). (TIFF) [file pone.0187721.s001.tiff]

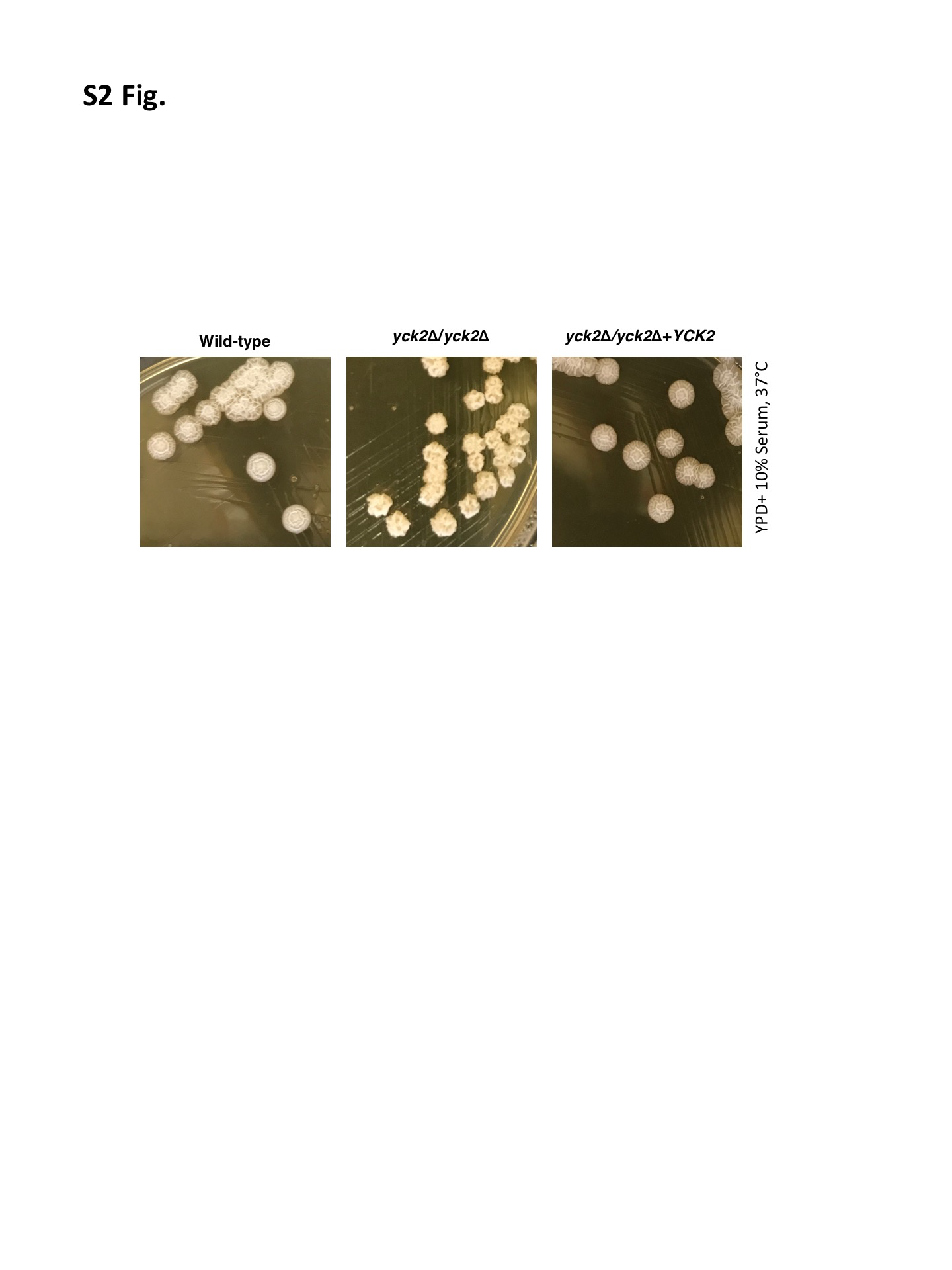

Supplement: S2 Fig — Colony morphologies of the wild-type (a), the yck2Δ/yck2Δ (b), and the yck2Δ/yck2Δ+YCK2 complemented (c) strains grown on YPD with 10% serum plate at 37°C for 2 days. (TIFF) [file pone.0187721.s002.tiff]

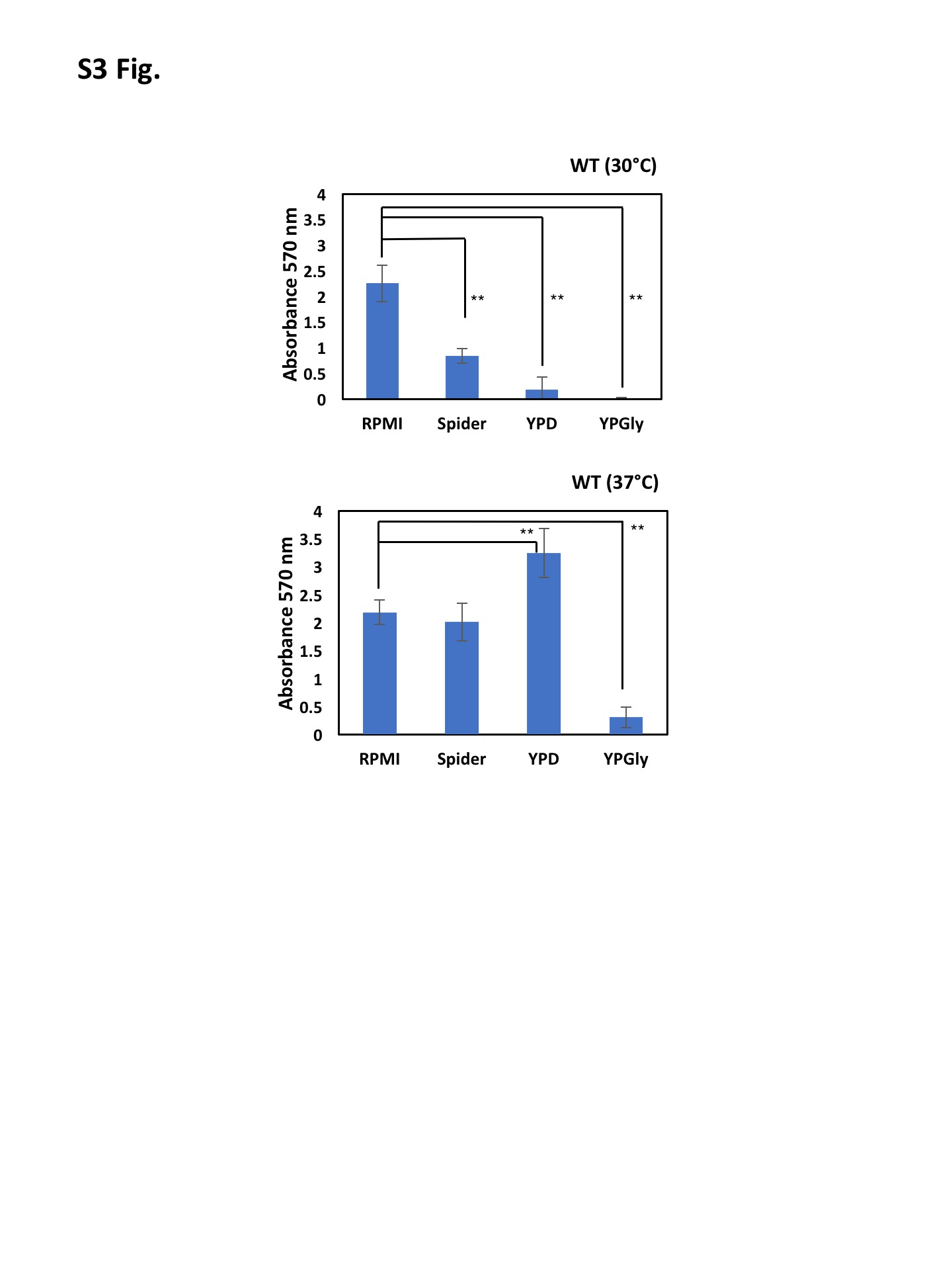

Supplement: S3 Fig — The extent of biofilm formation in RPMI1640, spider, YPD and YPglycerol media at 30°C and 37°C was tested by colormetric absorbance analysis at 595nm. (** p<0.01 in OneWay ANOVA with Dunnett’s test posthoc analysis). (TIFF) [file pone.0187721.s003.tiff]
