# Supplementary material for: Yeast casein kinase 2 governs morphology, biofilm formation, cell wall integrity, and host cell damage of Candida albicans
Source: PLoS One. 2017 Nov 6;12(11):e0187721. doi: 10.1371/journal.pone.0187721 (PMC5673188; doi:10.1371/journal.pone.0187721)
Supplement: S1 Table — (PDF) [file pone.0187721.s004.pdf]

S1 Table. Strains used in this study

Strain name	Genotype	Reference
SC5314	Wild-type	90
BWP17	*ura3Δ::λimm434::/ura3Δ::λimm434 arg4::hisG/arg4::hisG his1::hisG/his1::hisG*	80
DIC185	*ura3Δ::λimm434::URA3-IRO1/ura3Δ::λimm434 arg4::hisG::ARG4/arg4::hisG his1::hisG::HIS1/his1::hisG*	81
JJH34H	*ura3Δ::λimm434::/ura3Δ::λimm434 yck2-Tn7::UAU1/yck2-Tn7::URA3::pHIS1*	37
JAC1401U	*ura3Δ::λimm434::/ura3Δ::λimm434 yck2Δ::HIS1/yck2Δ::ARG4, his1/his1, arg4/arg4 URA3/ura3*	This study
JAC14010	*ura3Δ::λimm434::/ura3Δ::λimm434 yck2Δ::HIS1/yck2Δ::ARG4, his1/his1, arg4/arg4 ura3::URA3-YCK2/ura3*	This study
vps15 mut	*vps15Δ::HIS1/vps15Δ::ARG4 RP10::URA3 ura3Δ::λimm434/ ura3Δ::λimm434 arg4::hisG/arg4::hisG his1::hisG/his1::hisG*	91
