# Supplementary material for: Yeast casein kinase 2 governs morphology, biofilm formation, cell wall integrity, and host cell damage of Candida albicans
Source: PLoS One. 2017 Nov 6;12(11):e0187721. doi: 10.1371/journal.pone.0187721 (PMC5673188; doi:10.1371/journal.pone.0187721)
Supplement: S2 Table — (PDF) [file pone.0187721.s005.pdf]

S2 Table. Primers used in this study

Description	Primer Name	Sequence
**Strain construction**	YCK2KO-5	GATCTCCACTCTCTTCCTCCTCCTCCTTCTTTTCTTTTCTTTTCTTTTCTCCTTCTCCTTCTCCTTCTCCTTCGTTGAGTGTGGAATTGTGAGCGGATA
	YCK2KO-3	TGGAAATCCAAATTTATCATATCTTTGTATATCAAAAAAAACTTAACAAAAACACATAAAAATAGTAGCAAAATTCCAGTGCTTTCCCAGTCACGACGTT
	YCK2 Comp-5	GCATGCTAAATAATTGGTGGTGTGG
	YCK2 Comp-3	TGCAATTAGACCCTTTTCTTCC
	YCK2 Confirm-5	CTCTCTCTGTCTCTCTCTCTTAC
	YCK2 Confirm-3	GCTTGGCTTGAGCTTGTAATTG
**Transcription analysis**	ACT1-F	CTTCTCAATCTTCTGCCATTGA
	ACT1-R	TTCTGGACTCTGAATCTTTCG
	ALS3-F	TGCTCCATCGCAACTTCAA
	ALS3-R	GCGATTGAGATTGGTTGGTT
	BCR1-F	AATGCCTGCAGGTTATTTGG
	BCR1-R	TTTTAGGTGGTGGTGGCAAT
	CHS1-F	TGAGGACGAAGTTGCATTTG
	CHS1-R	CCAGCTATCTTTGCCCCATA
	CHS2-F	ATCGACTTGGGGGAAAGATT
	CHS2-R	TGCCAATAATGCTTGTGCTC
	CHS3-F	TGCCATGTCGTCTTTTCAAC
	CHS3-R	CATCACGAGCGGTCTCACTA
	CHS8-F	TGGAATCGGTGTTTGGATTT
	CHS8-R	CGGACCTTCTCCATTGACAT
	CPH1-F	AAAGGCAATACCGCAAACTG
	CPH1-R	CCGGTATTTCTGCTGGGTAA
	EFG1-F	CCCCCATACCTTCCAATTCT
	EFG1-R	TTGGTTGTTGCATTGTCGAT
	GSC1-F	GAACCTGGTTGCATTTCGTT
	GSC1-R	AGGTTGGGGAGTTGTAAGCA
	GSL1-F	TGTTGATATGGGGTGAAGCA
	GSL1-R	TGGGAAGATTGGGACCATTA
	GSL2-F	AGGTCTAACCGCTCATGGTG
	GSL2-R	TCGCTCAATTTGCATACAGC
	HGC1-F	GTATCGCTGGTTCTCGTGC
	HGC1-R	GACTCCACTCATAACACTACC
	HOG1-F	GGGACTGTTTTTGAAATCACC
	HOG1-R	TCTTTTTGCCAATACTGAGGTCA
	HRR25-F	CAACTCCCACCACAACAACA
	HRR25-R	TTGTGGAGGTTGTTGGGCTT
	HWP1-F	GCTGGCTCAAGTGGTGCTAT
	HWP1-R	GGTTGCATGAGTGGAACTGA
	MKC1-F	GTAAACGTGCTTTGCGTGAA
	MKC1-R	TCATGGGATTGGGGATAATG
	NRG1-F	CAACACCACCACCATATCCA
	NRG1-R	GGGTTGCAAGTGAGGTGATT
	RFG1-F	ATTCCTCGACCAAGAAATGC
	RFG1-R	GGGAAACTTCAGGGTTGGTT
	SUN41-F	AACCCTTTCCCTTCCATCTG
	SUN41-R	ACCAGAACCAGAACCACCAG
	TUP1-F	AAAGTCGACCTGCGAGGTAA
	TUP1-R	GCCCGACAAAATGTACTCGT
	UME6-F	TCATTCTGCTGATTTGGTCAT
	UME6-R	TTGCAGCAGCACTAACACTG
	YCK2-F	CAGCATGGAGGAAAACATGA
	YCK2-R	TCTTCTTCTTCGGCAACCATG
	YCK3-F	CCTTCTCAGGGACAAAGACG
	YCK3-R	ATGCTTGATGGGGTGAATTG
